# Efficacy and safety of intraperitoneal chemotherapy in patients with advanced gastric cancer: a cumulative meta-analysis of randomized controlled trials

**DOI:** 10.18632/oncotarget.20818

**Published:** 2017-09-11

**Authors:** Zheng He, Ting-Ting Zhao, Hui-Mian Xu, Zhen-Ning Wang, Ying-Ying Xu, Yong-Xi Song, Zhong-Ran Ni, Hao Xu, Song-Cheng Yin, Xing-Yu Liu, Zhi-Feng Miao

**Affiliations:** ^1^ Department of Radiation Oncology, First Hospital of China Medical University, Shenyang, Liaoning Province, China; ^2^ Department of Breast Surgery, First Hospital of China Medical University, Shenyang, Liaoning Province, China; ^3^ Department of Surgical Oncology, First Hospital of China Medical University, Shenyang, Liaoning Province, China; ^4^ School of Life Science, University of Technology Sydney, Ultimo, New South Wales, Australia; ^5^ Department of Medical Oncology, Shengjing Hospital of China Medical University, Shenyang, Liaoning Province, China

**Keywords:** gastric cancer, intraperitoneal chemotherapy, prognosis, meta-analysis

## Abstract

Even when a curative gastrectomy is conducted, the majority of advanced gastric cancer patients with invasion die due to peritoneal recurrence. We performed electronic searches to identify randomized controlled trials published through April 2017 evaluating the effect of intraperitoneal chemotherapy (IPC) on survival rates. We included 23 trials reporting data on 2,767 patients with advanced gastric cancer. Overall, we noted that patients who received IPC had a significantly increased 1-year survival rate, and the treatment effect of IPC on 1-year survival was most prominent in studies conducted in Japan or those with a mean age of less than 60 years. IPC was also associated with an increased incidence of 2-year survival rate, but it was not seen to have this effect in studies conducted in China or Australia or with a mean age greater than 60 years. Similarly, IPC associated with a significantly increased 3-year survival rate, but this difference was not detected in studies conducted in Austria or with a mean age greater than 60 years. IPC has no significant effect on the 5-year survival rate. Finally, IPC was associated with a lower risk of recurrence in patients with advanced gastric cancer. The findings of this study suggest that gastric cancer patients who receive IPC associate with increased 1-year, 2-year, and 3-year survival rates, but this does not extend out to a 5-year survival rate. IPC is also shown to play a protective role against the risk of recurrence in patients with advanced gastric cancer.

## INTRODUCTION

Gastric cancer is the fourth most common digestive cancer and the second leading cause of cancer-related deaths around the world [1, 2]. A high proportion of patients present with advanced stages at the time of diagnosis due to the lack of specific symptoms. Surgery remains the major curative treatment of choice for patients with gastric cancer, comprising of radical subtotal or total gastrectomy with D1–D2 lymph node dissection. Surgery is usually combined with systemic perioperative chemotherapy, which has demonstrated benefits of significantly increasing survival rate at different follow-up durations than in patients who received surgery alone [3, 4]. However, even in patients who received systemic chemotherapy, peritoneal dissemination is a major cause of gastric cancer recurrence due to tumor-cell spillage in perioperative period or during surgery [5].

The goal of treatments for peritoneal carcinomatosis is to heighten the concentration and amount of the drug in the peritoneum while reducing concentrations in the plasma. Previous trials have demonstrated the treatment effect of intraperitoneal chemotherapy (IPC) of colorectal origin, including for pseudomyxoma and mesothelioma [6–8]. However, the treatment effect of IPC in carcinomatosis of gastric origin remains disappointing and inconclusive. Several trials have indicated that IPC may increase survival rate in advanced gastric cancer [9–15], while the results of another trial showed adverse effects on survival rate [16]. Furthermore, most relevant trials have suggested that IPC has no significant effect on survival rate [17–31]. Clarifying any potential treatment effect of IPC in patients with advanced gastric cancer is particularly important, as it has not been definitively determined. Due to the potentially substantial implications that a proven clinical efficacy and safety of IPC in advanced gastric cancer would have, we undertook a systematic review and meta-analysis of all available RCTs to estimate the efficacy and safety of IPC in advanced gastric cancer. Furthermore, the treatment effect of IPC was compared among patients with different characteristics.

## RESULTS

### Literature search

**Figure 1:**
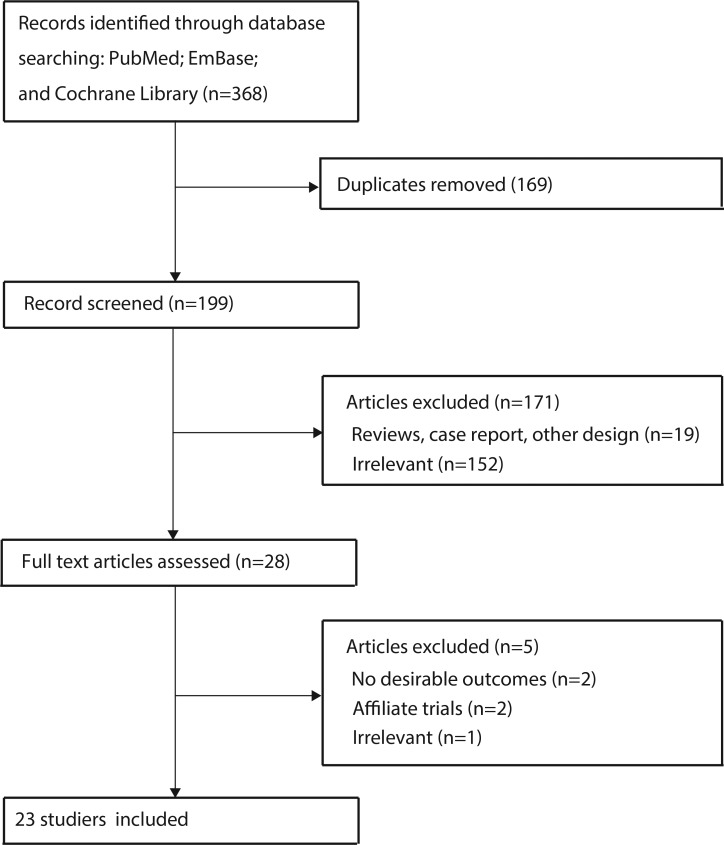
PRISMA flowchart of the selection of included studies

The results of the study-selection process are shown in Figure 1. Three hundred and sixty-eight articles were identified in our initial electronic search, of which 340 were excluded as duplicates or irrelevant studies. We retrieved the full text for the remaining 28 potentially eligible trials and, after detailed evaluations, twenty-three RCTs were selected for the final meta-analysis [9–31]. A manual search of the reference lists of these studies did not yield any new eligible studies. The general characteristics of the included studies were presented in Table 1.

**Table 1:** Baseline characteristic of studies included in the systematic review and meta-analysis

Author	Publication year	Country	Sample size	Mean age (yrs)	Percentage of male (%)	Disease status	Intervention	Follow-up (yrs)	Jadad scale
Koga [9]	1988	Japan	60	NA	70.2	I/II: 4.3%; III/IV: 95.7%	Mytomicin C	2.5	4
Hagiwara [17]	1992	Japan	49	54.3	71.4	I/II: 16.3%; III/IV: 83.7%	Mytomicin C	4.8	5
Hamazoe [18]	1994	Japan	82	59.9	68.3	I/II: 17.1%; III/IV: 82.9%	Mytomicin C	10.0	4
Fujimura [10]	1994	Japan	58	62.0	53.4	I/II: 34.5%; III/IV: 65.5%	Cisplatin	3.0	4
Sautner [19]	1994	Austria	67	62.9	NA	I/II: 0.0%; III/IV: 100.0%	Cisplatin	7.0	6
Takahashi [11]	1995	Japan	113	55.1	60.2	I/II: 11.5%; III/IV: 88.5%	Mitomycin C and activated carbon particles	3.5	5
Ikeguchi [12]	1995	Japan	174	61.8	61.5	I/II: 0.0%; III/IV: 100.0%	Mytomicin C	5.0	3
Rosen [20]	1998	Austria	91	NA	67.0	I/II: 0.0%; III/IV: 100.0%	Mitomycin C and activated carbon particles	2.7	5
Shimoyama [21]	1999	Japan	46	56.8	69.6	I/II: 45.7%; III/IV: 54.3%	Mytomicin C	6.0	3
Fujimoto [22]	1999	Japan	141	58.8	71.6	I/II: 17.0%; III/IV: 83.0%	Mytomicin C	10.0	3
Tan [23]	2000	China	51	51.2	54.9	I/II: 0.0%; III/IV: 100.0%	Mytomicin C	3.0	4
Yu [13]	2001	Korea	248	54.5	66.5	I/II: 37.5%; III/IV: 62.5%	Mitomycin C and 5-fluorouracil	5.0	4
Yonemura [24]	2001	Japan	139	59.5	59.7	I/II: 0.0%; III/IV: 100.0%	Mytomicin C and cisplatin	10.0	4
Zuo [25]	2004	China	82	52.6	58.5	I/II: 22.0%; III/IV: 78.0%	Mitomycin C, 5-fluorouracil, and cisplatin	3.0	2
Wei [26]	2005	China	156	56.0	67.9	I/II: 26.3%; III/IV: 73.7%	5-fluorouracil	3.0	4
Ding [27]	2007	China	78	53.6	78.2	I/II: 25.6%; III/IV: 74.4%	Cisplatin	3.0	4
Deng [28]	2009	China	85	52.5	77.6	I/II: 29.4%; III/IV: 70.6%	Mitomycin C and 5-fluorouracil	3.0	2
Kuramoto [29]	2009	Japan	88	64.9	45.5	I/II: 14.8%; III/IV: 85.2%	Cisplatin	5.0	7
Miyashiro [30]	2011	Japan	268	58.0	67.9	I/II: 0.0%; III/IV: 100.0%	Cisplatin	12.0	7
Yang [14]	2011	China	68	50.5	51.5	I/II: 0.0%; III/IV: 100.0%	Mytomicin C and cisplatin	5.0	6
Kang [31]	2014	Korea	521	54.5	67.4	I/II: 37.2%; III/IV: 62.8%	Cisplatin	6.0	3
Huang [16]	2014	China	42	57.1	59.5	I/II: 16.7%; III/IV: 83.3%	Cisplatin	4.0	3
Zheng [15]	2015	China	60	55.0	61.7	I/II: 0.0%; III/IV: 100.0%	5-fluorouracil	5.0	2

NA: not available.

### Study characteristics

The twenty-three included trials had studies involving a total of 2,767 advanced gastric cancer patients. The mean age for the patients was 50.5–64.9 years, and 42–521 patients were included in each trial. Eleven trials were conducted in Japan, 8 in China, 2 in Korea, and the remaining 2 in Austria. Trials investigating 1-year survival rate were available in all of the included trials, 2-year survival rates were available in 20 trials, 3-year survival rates in 21 trials, 5-year survival rates in 11 trials, and recurrence was reported upon in 14 trials. Seven trials used mytomicin C alone as the intraperitoneal drug, seven trials used mytomicin C combined with other drugs, seven trials used cisplatin alone, and the remaining two trials used 5-fluorouracil. Study quality was evaluated using the Jadad scale, with a trial scoring ≥ 4 regarded as being of high quality. Overall, two trials had a score of 7 [29, 30], 2 trials had a score of 6 [14, 19], 3 trials had a score of 5 [11, 17, 20], 8 trials had a score of 4 [9, 10, 13, 18, 23, 24, 26, 27], 5 trials had a score of 3 [12, 16, 21, 22, 31], and the remaining 3 trials had a score of 2 [15, 25, 28].

### 1-year survival rate

**Figure 2:**
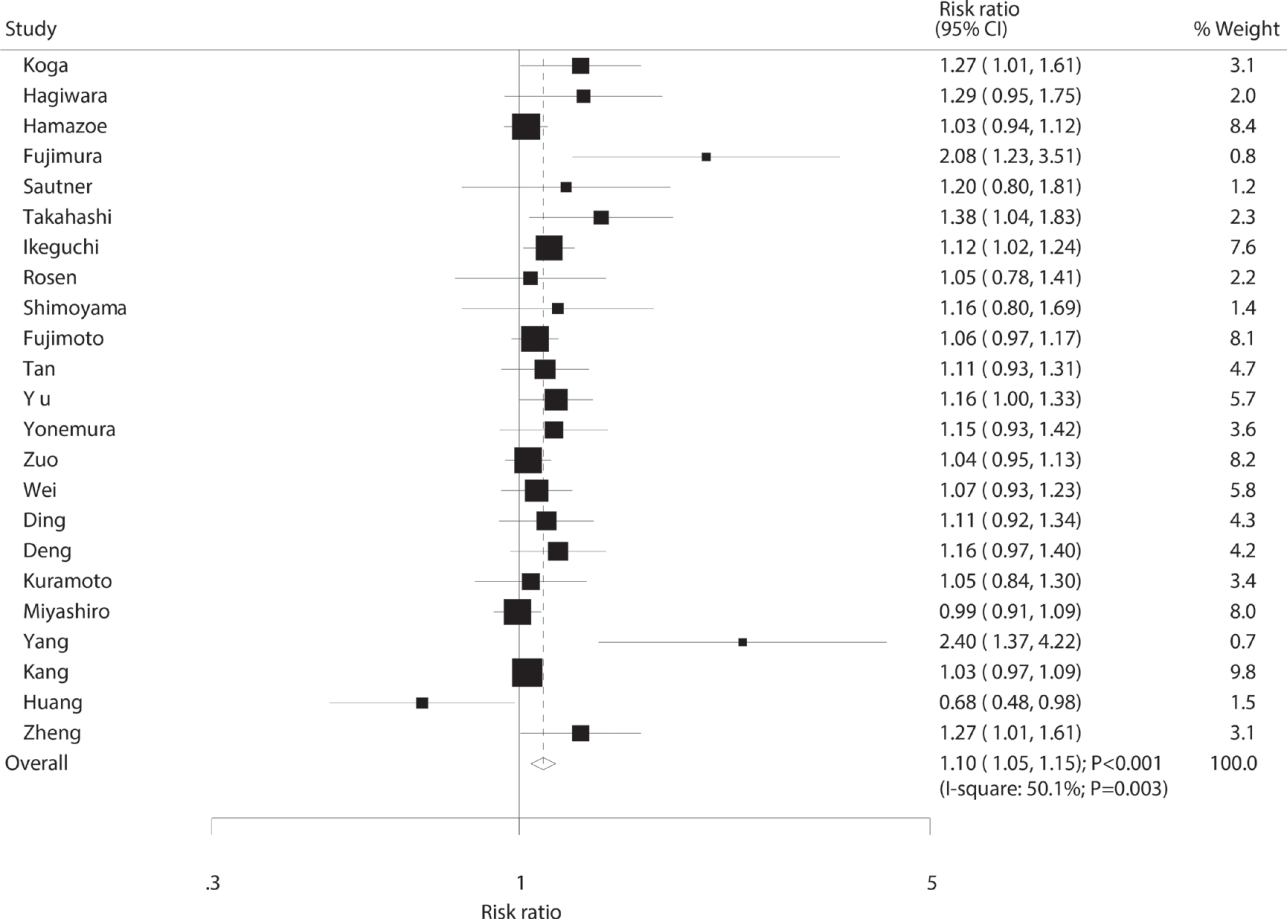
Effect of IPC on 1-year survival rate

Data for the effect of IPC on 1-year survival rate were available from 23 trials involving a total of 2,744 patients with advanced gastric cancer [9–31]. We noted that patients receiving IPC associated with an increased 1-year survival rate (RR: 1.10; 95%: 1.05–1.15; *P* < 0.001; Figure 2), with substantial heterogeneity observed (I^2^ = 50.1%; *P* = 0.003). A sensitivity analysis indicated that the results were not affected by the sequential exclusion of any particular trial from all pooled analyses (Supplementary Table 1).

### 2-year survival rate

**Figure 3:**
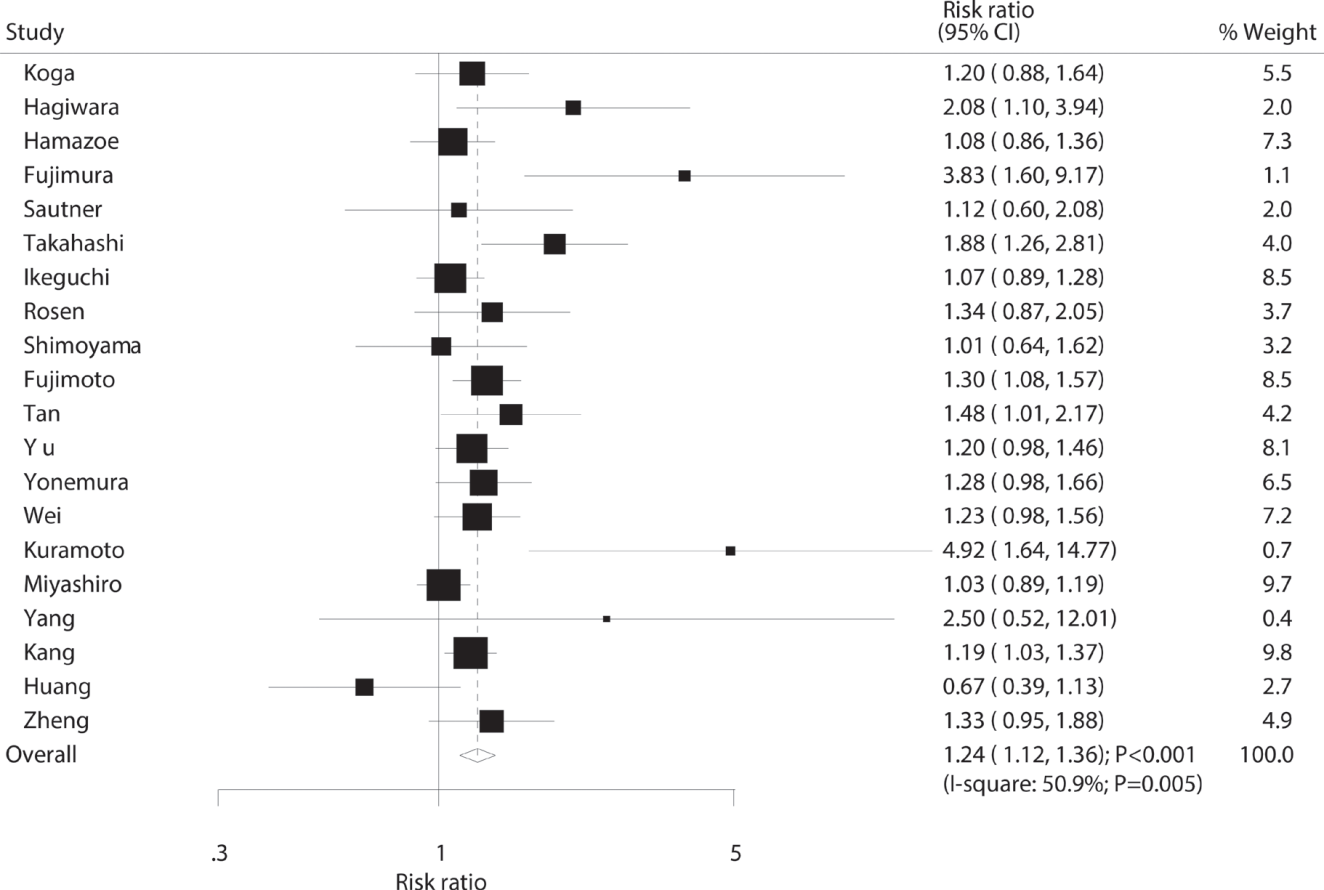
Effect of IPC on 2-year survival rate

Data for the effect of IPC on 2-year survival rate were available from 20 trials involving a total of 2,499 patients with advanced gastric cancer. The pooled RR showed a 24% increase in 2-year survival rate, an association which was statistically significant (RR: 1.24; 95% CI: 1.12–1.36; *P* < 0.001; Figure 3). Potential evidence of significant heterogeneity was observed (I^2^ = 50.9%; *P* = 0.005). After a sensitivity analysis was conducted, the conclusion was not seen to be affected by the exclusion of any specific study (Supplementary Table 2).

### 3-year survival rate

**Figure 4:**
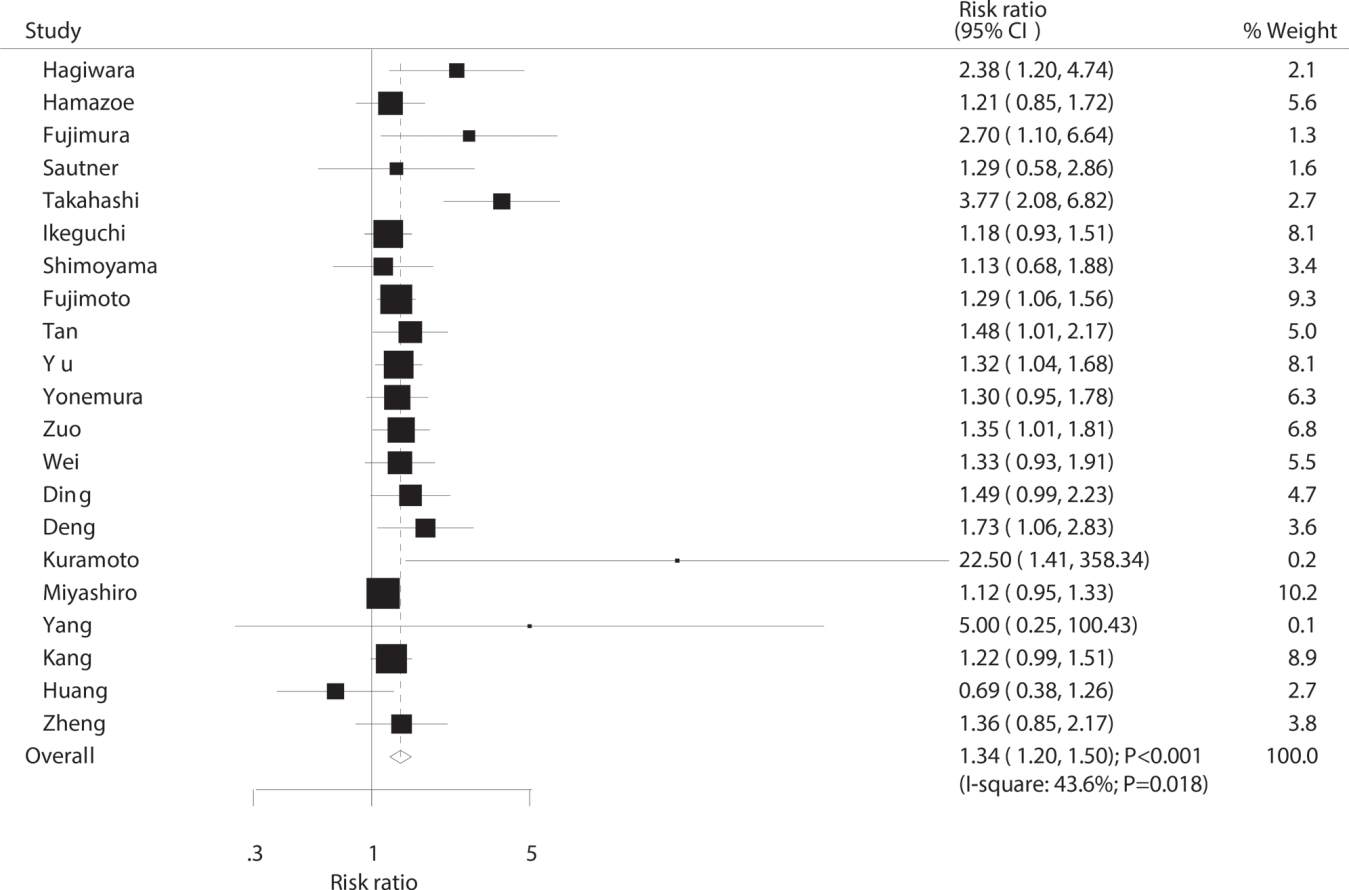
Effect of IPC on 3-year survival rate

Data for the effect of IPC on 3-year survival rate were available from 21 trials involving a total of 2,593 patients with advanced gastric cancer. The summary RR indicated that IPC significantly increased the incidence of 3-year survival in patients with advanced gastric cancer (RR: 1.34; 95% CI: 1.20–1.50; *P* < 0.001; Figure 4). Although substantial heterogeneity was observed in the magnitude of the effect across the studies (I^2^ = 43.6%; *P* = 0.018), the conclusion was not affected by the exclusion of any specific study (Supplementary Table 3).

### 5-year survival rate

**Figure 5:**
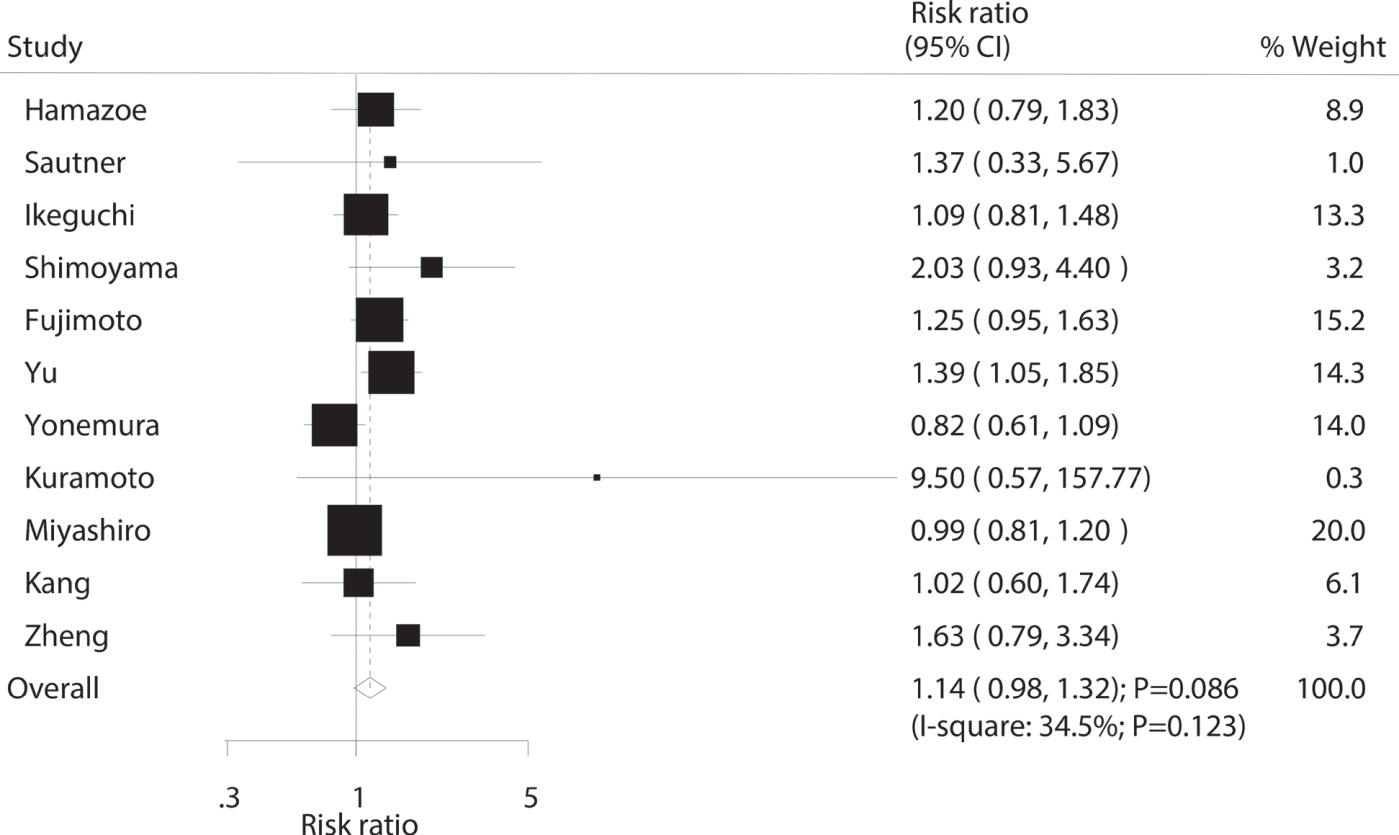
Effect of IPC on 5-year survival rate

Data for the effect of IPC on 5-year survival rate were available from 11 trials involving a total of 1,834 patients with advanced gastric cancer. The summary RR showed that IPC had no association with 5-year survival rate (RR: 1.14; 95% CI: 0.98-1.14; *P* = 0.086; Figure 5), and moderate heterogeneity was seen (I^2^ = 34.5%; *P* = 0.123). Following the sensitivity analysis, we excluded the study by Yonemura et al. which specifically included patients with longer follow-up duration, which may have completed follow-up records. After this exclusion, we could conclude that IPC significantly increased the incidence of 5-year survival rate by 18% (RR: 1.18; 95% CI: 1.03–1.34; *P* = 0.014; Supplementary Table 4).

### Recurrence

**Figure 6:**
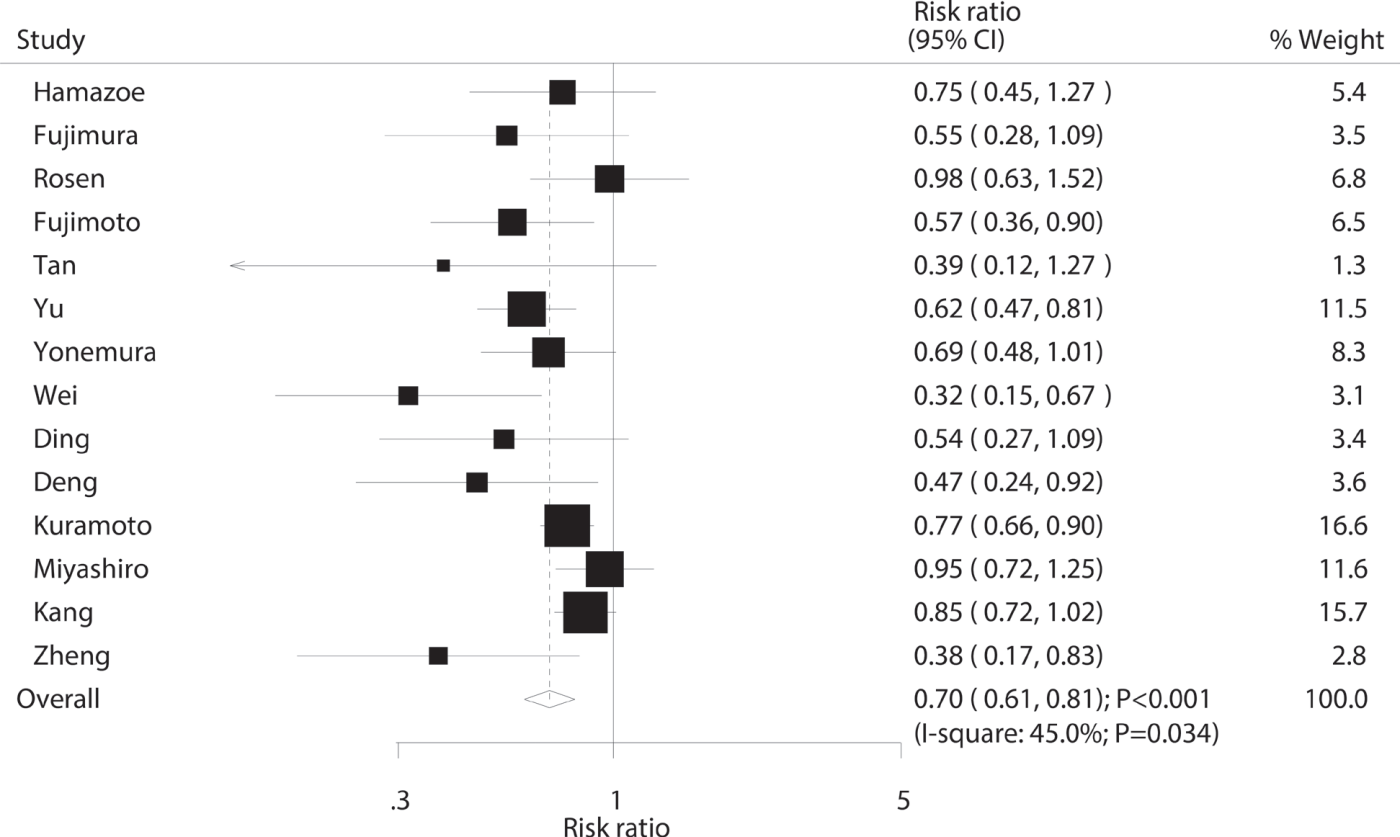
Effect of IPC on the risk of recurrence

Data for the effect of IPC on the incidence of recurrence were available from 14 trials involving a total of 2,030 patients with advanced gastric cancer. Overall, IPC reduced the risk of recurrence by 30%, with the result shown to be statistically significant (RR, 0.70; 95% CI: 0.61–0.81; *P* < 0.001; Figure 6). Although substantial heterogeneity was detected across the included trials, the results were not affected by sequential exclusion of each trial from all pooled analyses (I^2^ = 45.0%; *P* = 0.034; Supplementary Table 5).

### Cumulative meta-analysis

The findings of the cumulative meta-analysis for 1-year survival rate are presented in Supplementary Figures 1–5. We noted that the treatment effect of IPC on 1-year survival rate is variable before 1994, yet the overall effect was associated with statistically significant improvement. Similarly, statistically significant improvements persisted in the cumulative meta-analyses for 2-year and 3-year survival rates. However, we noted the cumulative meta-analysis for 5-year survival was associated with statistical significance only if combined to Fujimoto et al and Yu et al's study, and the summary results indicated that IPC has no significant effect on 5-year survival rate in studies completed after 2001. Finally, the treatment effect of IPC showed consistently reduced risk of recurrence in studies completed after 1999.

### Meta-regression

Heterogeneity testing for the analyses showed *P* < 0.10 for 1-year survival rate, 2-year survival rate, 3-year survival rate, and recurrence. We concluded that heterogeneity was statistically significant in the overall analysis and conducted a meta-regression analysis for survival rates at different stages and recurrence that included publication year, sample size, mean age, percentage male, percentage III/IV gastric cancer, and duration of follow-up (Supplementary Figures 6–18). Overall, we noted that publication year (*P* = 0.143), sample size (*P* = 0.256), mean age (*P* = 0.768), percentage male (*P* = 0.699), percentage III/IV gastric cancer (*P* = 0.662), and duration of follow-up (*P* = 0.142) were not significant factors contributing to the association between IPC and 1-year survival rate. Nor did these factors affect IPC effect on 2-year survival rate or 3-year survival rate. Percentage of stage III/IV gastric cancer (*P* = 0.033) was seen to contribute to the association between IPC and 5-year survival rate, while no other factors contributed a significant effect (Supplementary Figures 19–24). Finally, these factors did not bias the effect of IPC on the risk of recurrence (Supplementary Figures 25–35).

### Subgroup analysis

Subgroup analyses were conducted for survival rates at different stages and recurrence to minimize heterogeneity among the included trials and to evaluate the efficacy and safety of IPC in specific patient subsets (Supplementary Table 6). First, we noted that IPC has no significant effect on 1-year survival rate if the study was conducted in Korea, China, or Austria, or if the mean age of patients was greater than 60 years. Second, there was no significant effect of IPC on 2-year survival rate if the study was conducted in China or Austria, or if the mean age of patients was greater than 60 years. Third, IPC had no effect on 3-year survival rate when the study was conducted in Austria or if the mean age of patients was greater than 60 years. Fourth, patients who received IPC were associated with an increased incidence of 5-year survival if the study was published before 2000, if it had a sample size of less than 100, or if the percentage of III/IV gastric cancer was less than 90%. Finally, IPC had no significant effect on recurrence when the study was conducted in Korea or Austria, the mean age of patients was greater than 60 years, or the percentage of III/IV gastric cancer was greater than 90%.

### Publication bias

**Figure 7:**
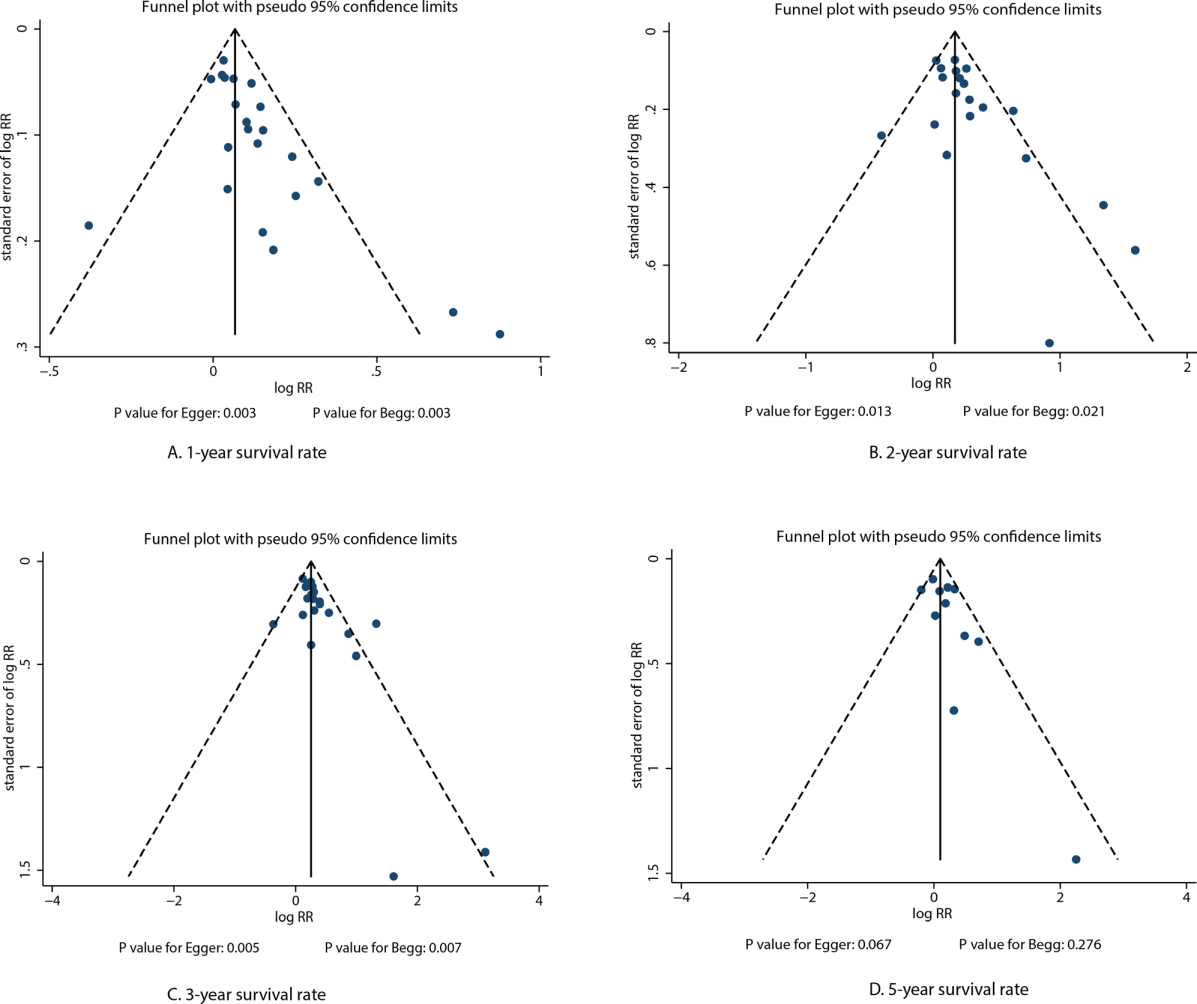
Funnel plots for survival rate at different stages

A review of the funnel plots could not rule out the potential for publication bias on survival rates at different stages. The Egger's [40] and Begg's test [41] results showed significant publication bias for 1-year survival rate (*P* value for Egger: 0.0.003; *P* value for Begg: 0.003; Figure 7A), 2-year survival rate (*P* value for Egger: 0.013; *P* value for Begg: 0.021; Figure 7B), and 3-year survival rate (*P* value for Egger: 0.005; *P* value for Begg: 0.007; Figure 7C). No evidence of publication bias was shown for 5-year survival rate (*P* value for Egger: 0.067; *P* value for Begg: 0.276; Figure 7D). Although there was potential for publication bias for the 1-year, 2-year, and 3-year survival rates, after adjusted by using trim and fill methods [42], we noted IPC was associated with an increased incidence of 1-year (RR: 1.06; 95% CI: 1.01–1.11; *P* = 0.030), 2-year (RR: 1.19; 95% CI: 1.07–1.33; *P* = 0.001), and 3-year survival rates (RR: 1.34; 95% CI: 1.20–1.49; *P* < 0.001).

## DISCUSSION

The objective of this meta-analysis was to determine the efficacy and safety of IPC in patients with advanced gastric cancer. Twenty-three RCTs were included involving 2,767 patients. The findings suggested that IPC was associated with an increased incidence of 1-year, 2-year, and 3-year survival rate, while it had little or no significant effect on the incidence of 5-year survival rate. Furthermore, patients with advanced gastric cancer who received IPC had a significantly reduced risk of recurrence. This treatment effect on survival rate at different stages and recurrence might be biased by country, mean age, and percentage of III/IV gastric cancer. These results could help to better define the efficacy and safety of IPC, and could also help physicians select the appropriate approach for treating patients with advanced gastric cancer.

A previous meta-analysis suggested that steroid therapy was associated with increased 1, 2, and 3-year overall survival in patients with advanced gastric cancer, while no significant difference between IPC plus surgery and surgery alone was noted for the 5-year survival rate [43]. They further suggested that IPC was associated with significantly improved 2 and 3-year mortality in patients with nodal invasion, and improved 1 and 2-year mortality in patients with serosal infiltration. In addition, patients who received IPC might see beneficial effects on peritoneal recurrence and distant metastasis. However, this study faced criticism because the treatment effects were not analyzed in specific sub-populations and they did not include sufficient information to provide strong evidence. Another important meta-analysis based on RCTs suggested that IPC may benefit patients after curative resection for locally advanced gastric cancer, and it was associated with greater protective effects to patients when combined with hyperthermia or activated carbon particles [44]. The inherent limitation of this previous review was that a smaller number of trials was included than was needed to show a clinical benefit in patients with specific characteristics. Therefore, we conducted a comprehensive quantitative meta-analysis of RCTs to evaluate the efficacy and safety of IPC in advanced gastric cancer.

Our summary results indicate that IPC is associated with increased 1, 2, and 3-year survival rates. However, as presented in Figures 2, 3, 4, we note that most included trials suggested no significant difference between IPC plus surgery and surgery alone. A possible reason for this could be that most trials included in this study had small sample sizes and were designed with other outcomes as their primary end points, so they might not have had adequate power to detect a potential clinically relevant difference in advanced gastric cancer. Furthermore, we saw no significant effect of IPC on 5-year survival rate, while Yu et al suggested that surgery and postoperative IPC significantly improve 5-year survival rate compared to surgery alone, with most treatment effects attributed to patients with I and II gastric cancer [12]. This study had a lower percentage of III/IV than other studies, which might have contributed to this significant treatment effect if the survival rate was higher in patients with I and II gastric cancer, and the study size was too small to accurately detect the treatment effect.

Our cumulative meta-analyses suggest that IPC has a significant effect on 1, 2, and 3-year survival rate if enough trials are summarized to provide sufficient statistical power. However, the proposed significant protective effect of IPC for 5-year survival rate has been refuted by the trial conducted by Yonemura et al. [24]. This trial specifically suggested that IPC reduced the incidence of 5-year survival rate by 18% (95% CI: 0.61–1.09), though this reduction was not statistically significant. A possible reason for this could be that this study randomly divided patients into groups receiving chemohyperthermia peritoneal perfusion, chemohyperthermia, or surgery alone, and different treatment regimens and controls might affect the 5-year survival rate. Further, number of included trials were variable among survival rate at different follow-up times, which might affect the treatment effect of IPC on 5-year survival rate in patients with advanced gastric cancer.

Subgroup analyses suggested that IPC had no effect on 1, 2, and 3-year survival rate based on country (Korea, China, or Austria), and when the mean age was greater than 60 years. This could be due to the higher incidence of gastric cancer and stellar treatment strategies in Japan, which contributed to higher sample sizes to acquire enough statistical power to see higher survival rates at different stages. Further, older patients with advanced gastric cancer are associated with poor prognosis, so the therapeutic effect might have contributed to this lack of significant difference. In addition, IPC was seen to have a protective effect on 5-year survival rate if the study was published before 2000, with a sample size less than 100, or if the percentage of III/IV gastric cancer was less than 90%. This indicates that the treatment effect of IPC plays greater effect in patients with stage I/II gastric cancer than in patients with stage III/IV gastric cancer. Further, the procedures in surgery are developed and variable, which might affect the treatment effect of IPC in advanced gastric cancer. In addition, IPC had no effect on recurrence if the study was conducted in Korea or Austria, with a mean age greater than 60 years, or with a percentage of III/IV gastric cancer greater than 90%. However, these conclusions may be unreliable since smaller cohorts were included in each subset. Therefore, we just presented a relative result and provided a synthetic and comprehensive review.

Three strengths of our meta-analysis should be highlighted. First, only prospective RCTs were included, which should eliminate confounders inherent to observational studies. Second, the large sample size allowed us to quantitatively evaluate the efficacy and safety of IPC in the treatment of advanced gastric cancer. Third, the treatment effect of IPC was analyzed in specific sub-populations to help physicians decide whether IPC is the optimal treatment for patients with different characteristics.

The limitations of our study should be mentioned: (1) different types and doses of chemotherapy regimens used between studies might result in bias; (2) differences in diagnosis and reporting for gastric cancer might have contributed to the differences in some trials; (3) several trials with lower study quality were included, which might bias the results; (4) the procedures in surgery are differ for the developed ranged 1988 to 2015, which affect the survival rate in patients with advanced gastric cancer; and (5) the analysis used pooled data (individual data were not available), restricting us from performing a more detailed relevant analysis to obtain more comprehensive results.

The findings of this study suggest that IPC is associated with higher 1, 2, and 3-year survival rates, whereas it has no significant effect on 5-year survival rate. Furthermore, IPC significantly reduces the risk of recurrence in patients with advanced gastric cancer. The findings of the subgroup analyses suggest that country, mean age, and gastric cancer stage potentially modulate the treatment effect of IPC. Future studies should focus on patients with different cancer stages and evaluate dosage, treatment regimens, and measurement effects to analyze the treatment effect of IPC in patients with specific characteristics.

## MATERIALS AND METHODS

### Data sources, search strategy, and selection criteria

This review was conducted and reported according to the Preferred Reporting Items for Systematic Reviews and Meta-Analysis Statement issued in 2009 [32] (Checklist S1). PubMed, EmBase, and the Cochrane library were searched for articles published from the initial use of steroid therapy in critical illness up to April 2017, using the keywords “intraperitoneal” AND “chemotherapy” AND (“stomach” OR “gastric”) AND (“cancer” OR “carcinosis” OR “tumor” OR “carcinoma” OR “neoplasm”) AND (“randomized controlled trials”). The search had no restrictions placed on language or publication status. The details of search strategy in PubMed are presented in Supplementary Figure 36. We also conducted manual searches of reference lists from all relevant original and review articles to identify additional eligible trials. The title, methods, disease status, study design, intervention, control, and outcomes within these trials were used to identify relevant studies.

Literature retrieval was performed in duplicate by two independent reviewers. A study was eligible for inclusion if the following criteria were met: (1) the study had a RCT design; (2) the trial compared IPC plus surgery with surgery alone in treatment of patients with advanced gastric cancer; (3) the study reported at least one of the following outcomes: survival rate at different stages (1-year, 2-year, 3-year, 5-year), or recurrence. For trials without adequate published data, we contacted the authors to get the unpublished results. If the author could not provide the necessary data, these trials were excluded.

### Data collection and quality assessment

Data extraction and assessment were performed independently by two reviewers. Publication information (first author's name, publication year), characteristics of patients (country, sample size, mean age, percentage male, disease status, intervention, follow-up duration periods), and outcomes (survival rate at different stages, recurrence) were extracted. Any inconsistency was settled by a third reviewer for consensus. Furthermore, two reviewers independently evaluated the quality of trials using the revised Jadad guidelines [33], which is based on randomization, concealment of the treatment allocation, blinding, completeness of follow-up, and the use of intention-to-treat analysis. The five-subscales questionnaire produces a total score ranging from 0 (worst) to 7 (best). In case of a disagreement, a consensus was reached after group discussion.

### Statistical analysis

The efficacy and safety of IPC in the treatment of advanced gastric cancer was evaluated on the basis of multiple events and sample sizes in each group, as published in each individual trial. Pooled relative risks (RRs) and 95% confidence intervals (CIs) were calculated using the random effects model for IPC plus surgery versus surgery alone [34, 35]. In the cumulative meta-analysis, outcome data for survival rate at different stages or recurrence from all available trials were included sequentially according to the year in which they first became available. Heterogeneity among trials was investigated using the Q statistic, and *P* values less than 0.10 were indicative of significant heterogeneity [36]. Potential sources of heterogeneity in the estimates of the treatment effect on survival rate at different stages and recurrence were explored using univariate meta-regression [37] (for publication year, sample size, mean age, percentage male, percentage III/IV gastric cancer, and duration of follow-up). Subgroup analyses were conducted based on publication year, country, sample size, mean age, percentage male, percentage III/IV gastric cancer, and follow-up duration. *P* values for heterogeneity between subgroups were also evaluated via the Chi-square test [38]. Sensitivity analyses were performed by sequentially removing each individual trial from the meta-analysis [39]. Publication bias was evaluated qualitatively using visual inspections of funnel plots, and the Egger's [40] and Begg's [41] tests were used to quantitatively assess publication bias. All reported *P* values were two-sided, and *P* values less than 0.05 were regarded as statistically significant for all included trials. Statistical analyses were conducted using STATA software (version 10.0; Stata Corporation, College Station, TX, USA).

## SUPPLEMENTARY MATERIALS FIGURES AND TABLES




