# Supplementary material for: Efficacy and safety of intraperitoneal chemotherapy in patients with advanced gastric cancer: a cumulative meta-analysis of randomized controlled trials
Source: Oncotarget. 2017 Sep 11;8(46):81125–36. doi: 10.18632/oncotarget.20818 (PMC5655267; doi:10.18632/oncotarget.20818)
Supplement: Supplementary file 1 [file oncotarget-08-81125-s001.pdf]

Efficacy and safety of intraperitoneal chemotherapy in patients with advanced gastric cancer: a cumulative meta-analysis of randomized controlled trials

SUPPLEMENTARY MATERIALS

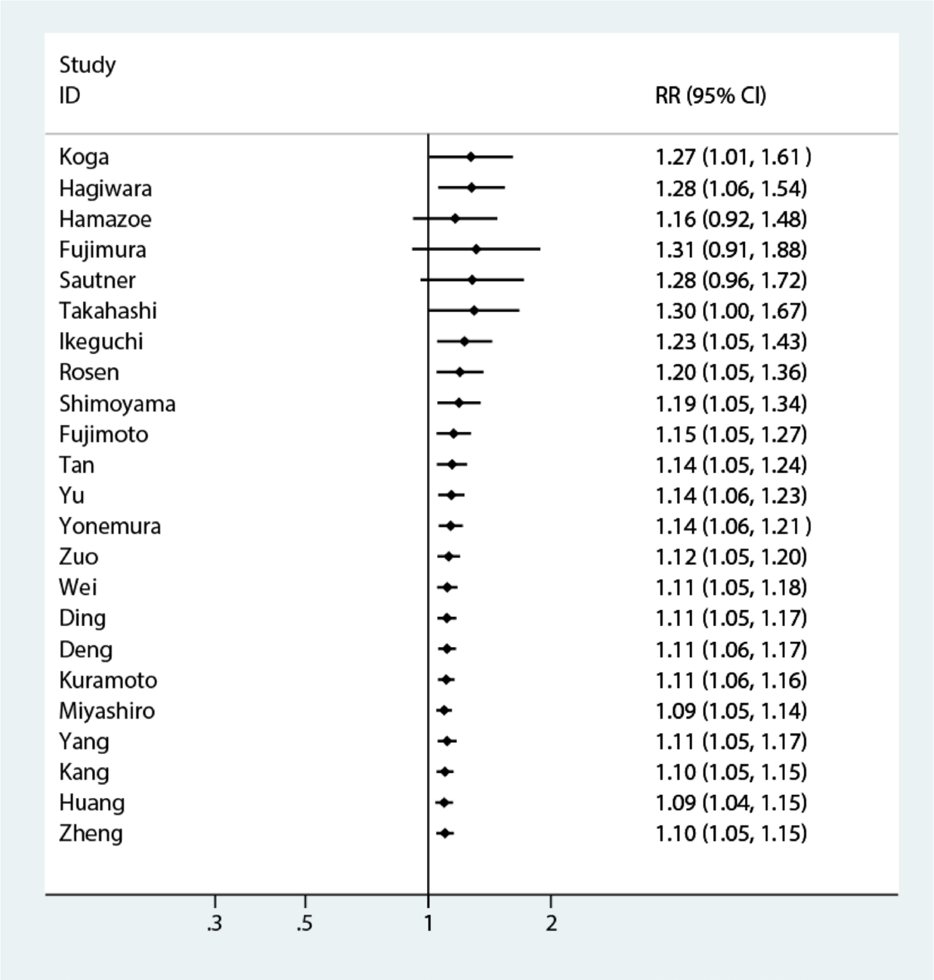

Supplementary Figure 1: Cumulative meta-analysis of the IPC on 1-year survival rate.

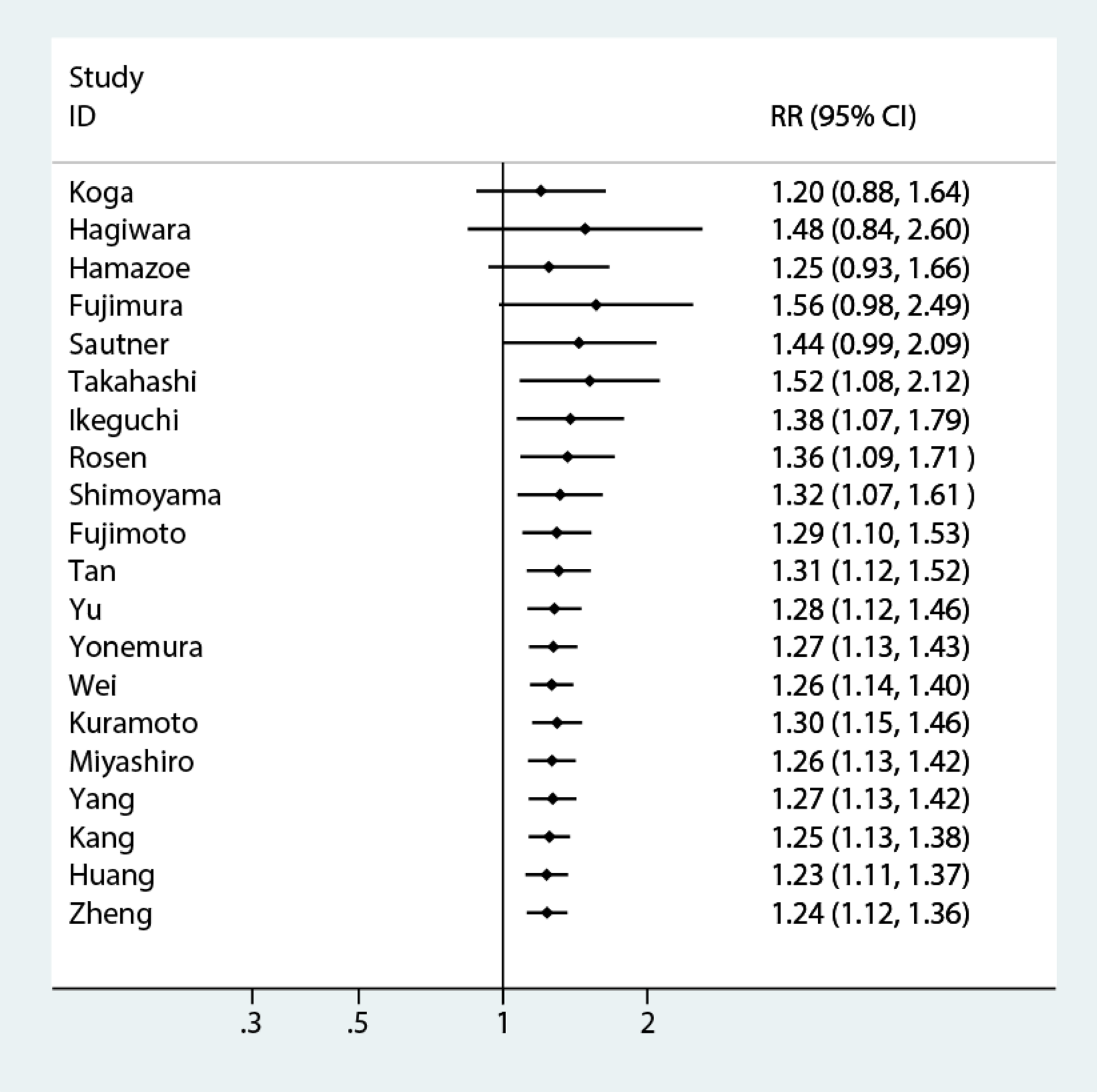

Supplementary Figure 2: Cumulative meta-analysis of the IPC on 2-year survival rate.

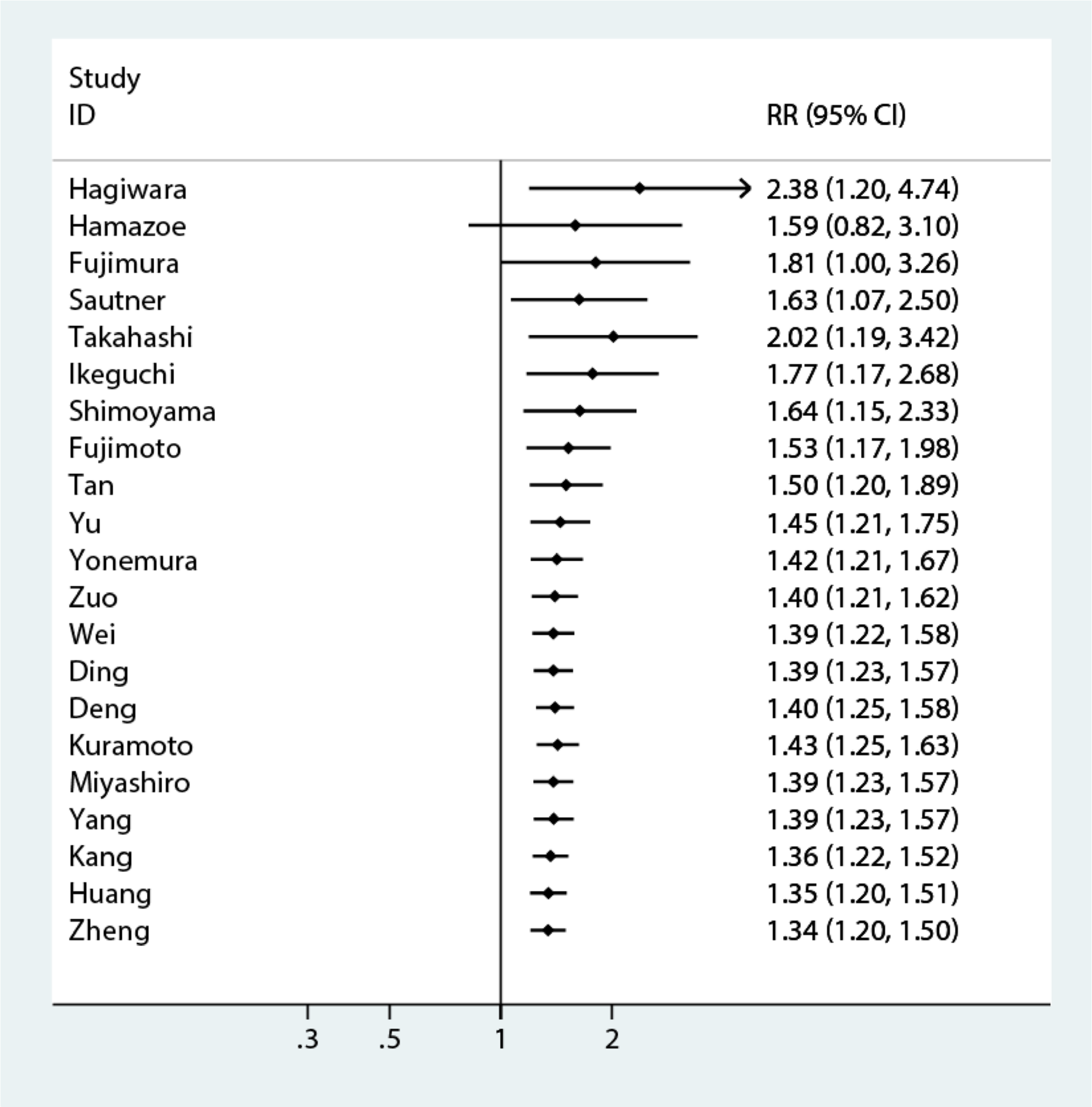

Supplementary Figure 3: Cumulative meta-analysis of the IPC on 3-year survival rate.

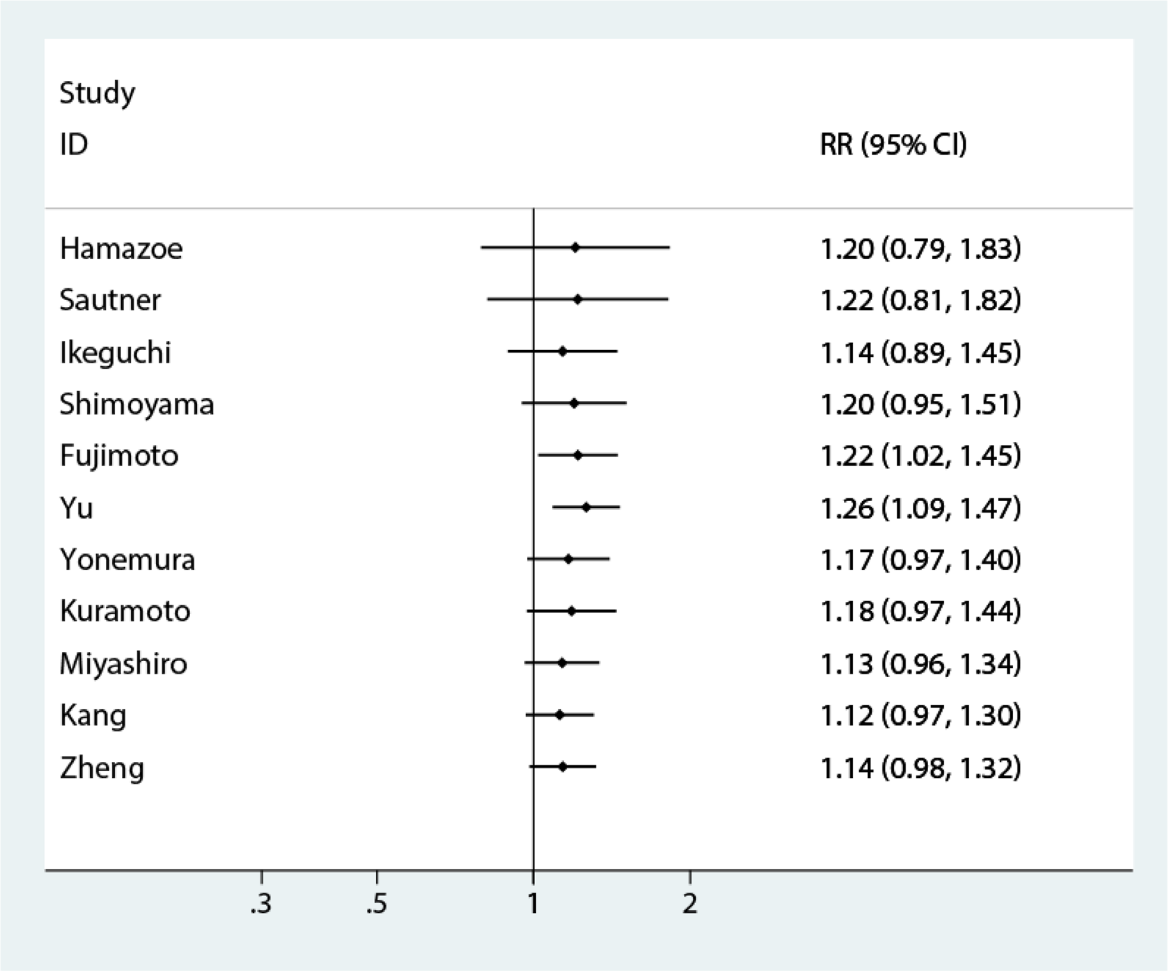

Supplementary Figure 4: Cumulative meta-analysis of the IPC on 5-year survival rate.

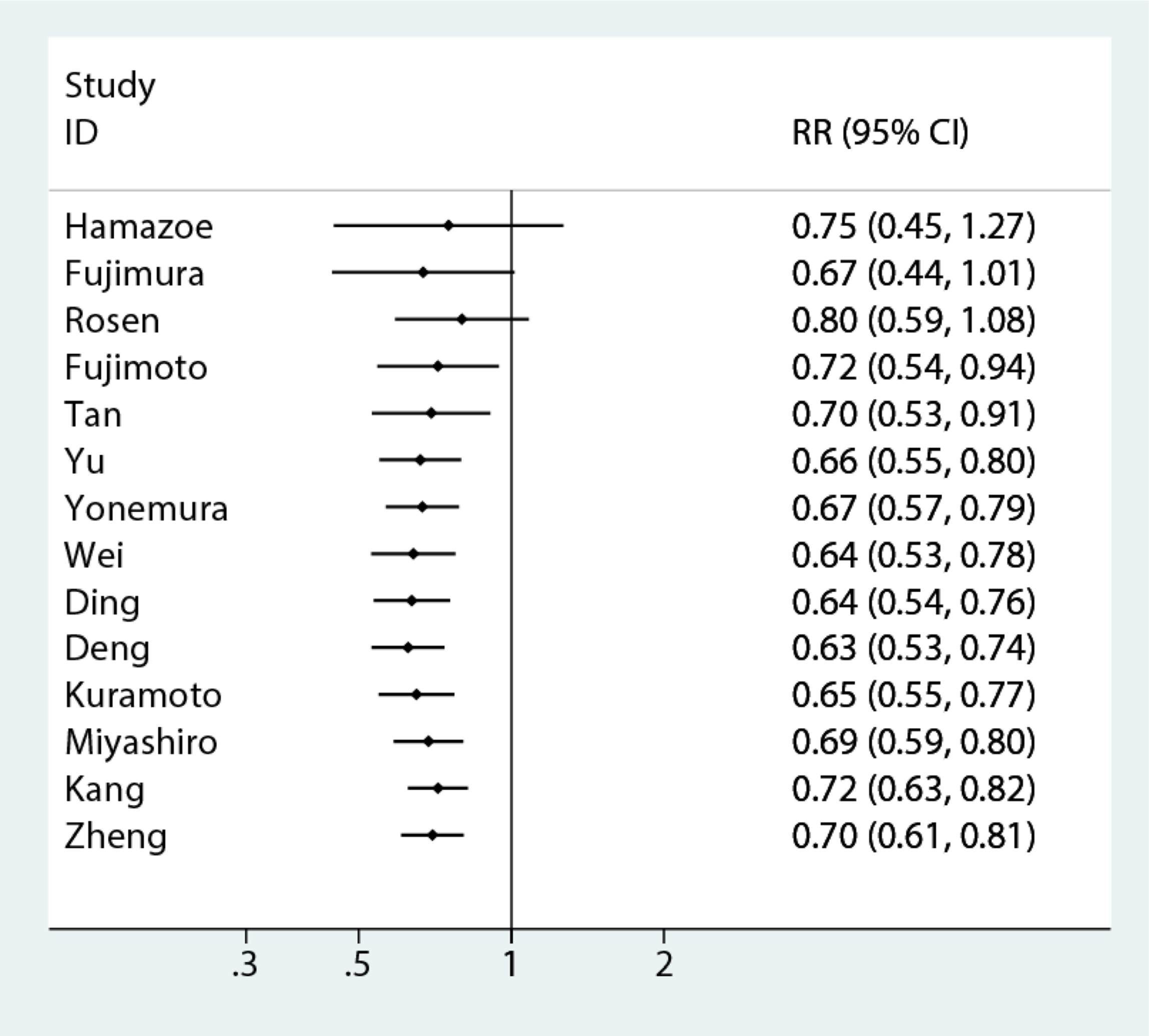

Supplementary Figure 5: Cumulative meta-analysis of the IPC on recurrence.

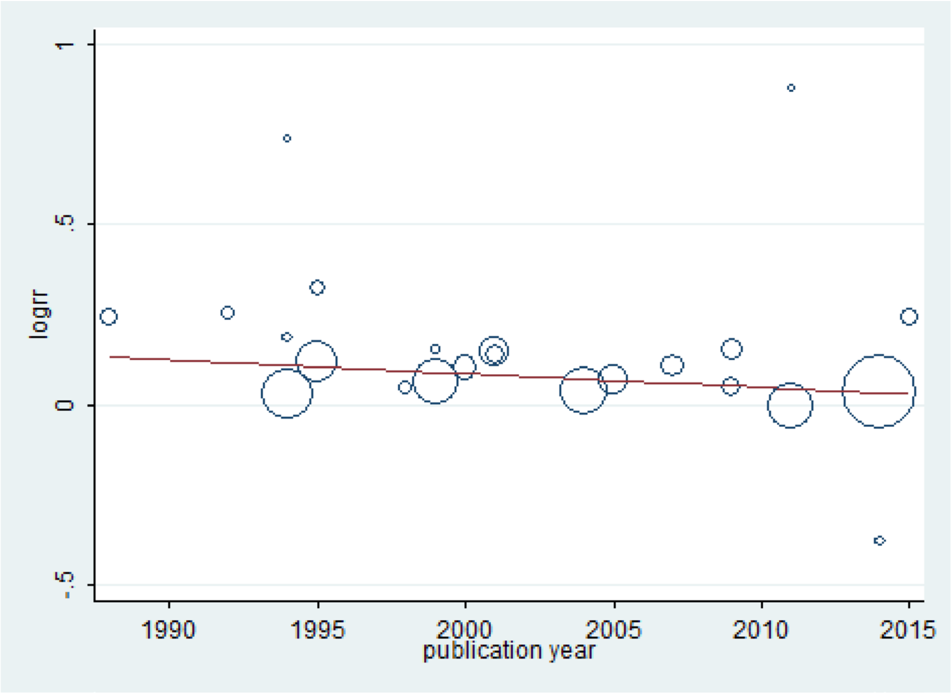

Supplementary Figure 6: Meta-regression for 1-year survival rate based on publication year (*P* = 0.143).

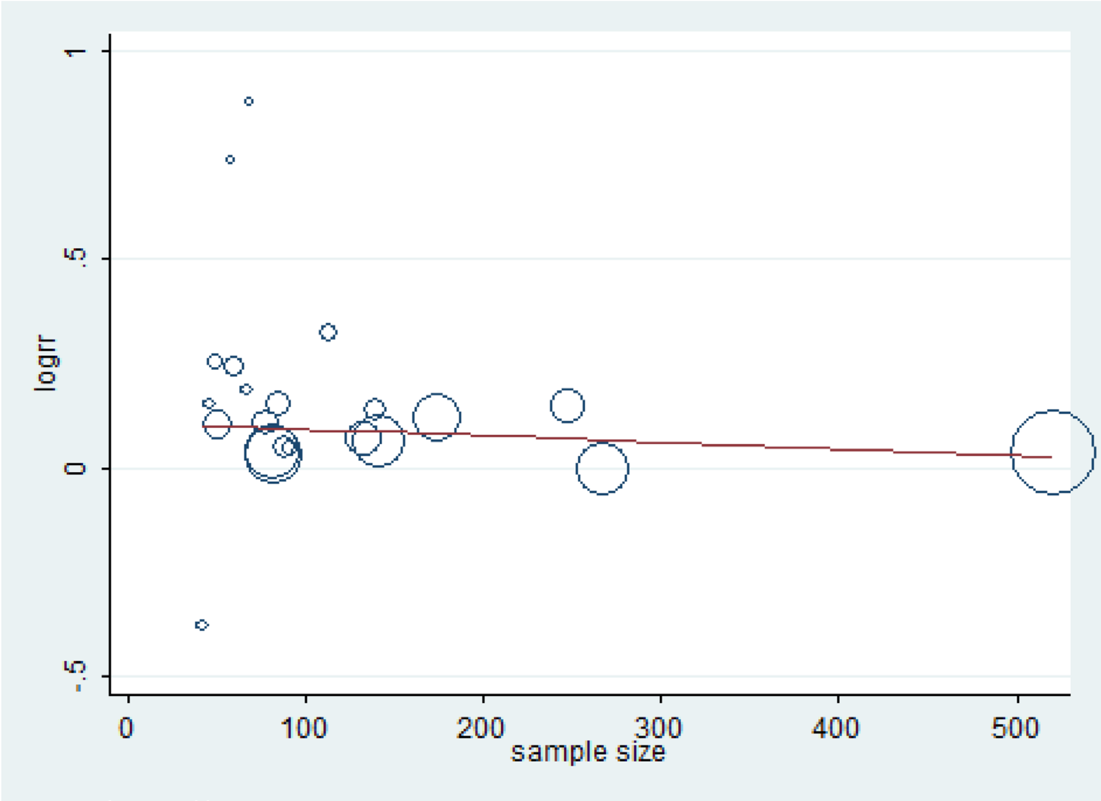

Supplementary Figure 7: Meta-regression for 1-year survival rate based on sample size (*P* = 0.256).

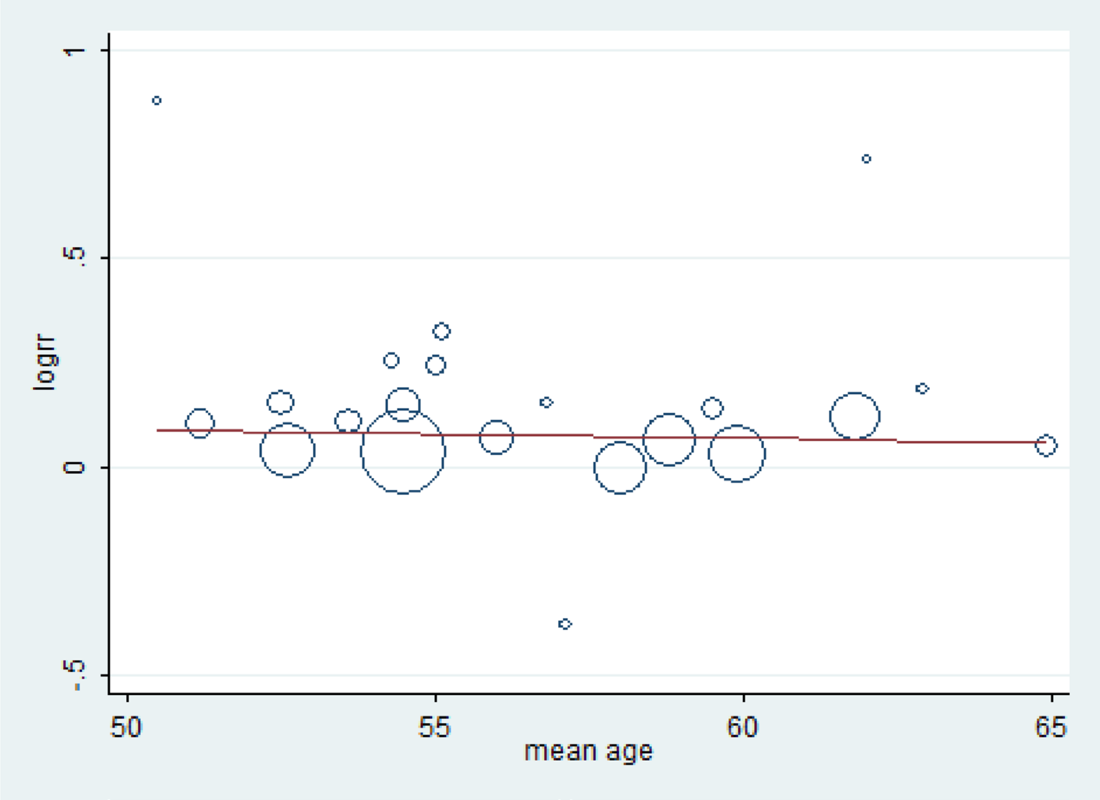

Supplementary Figure 8: Meta-regression for 1-year survival rate based on mean age (*P* = 0.768).

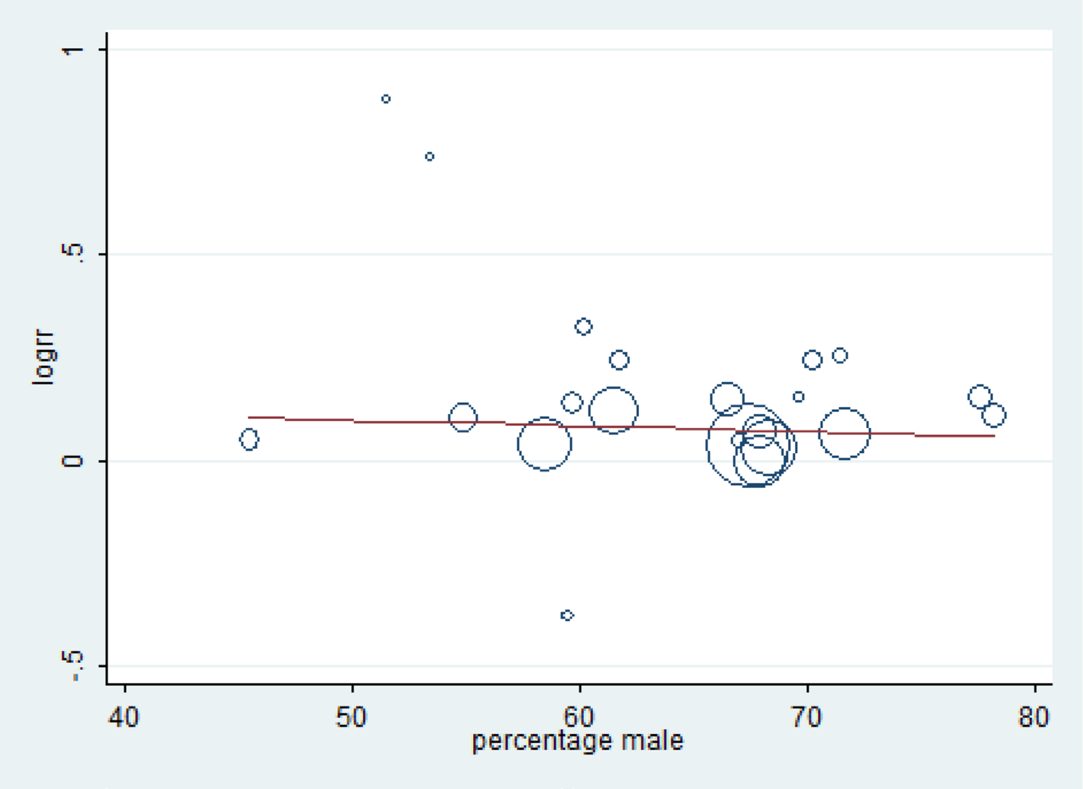

Supplementary Figure 9: Meta-regression for 1-year survival rate based on percentage male (*P* = 0.699).

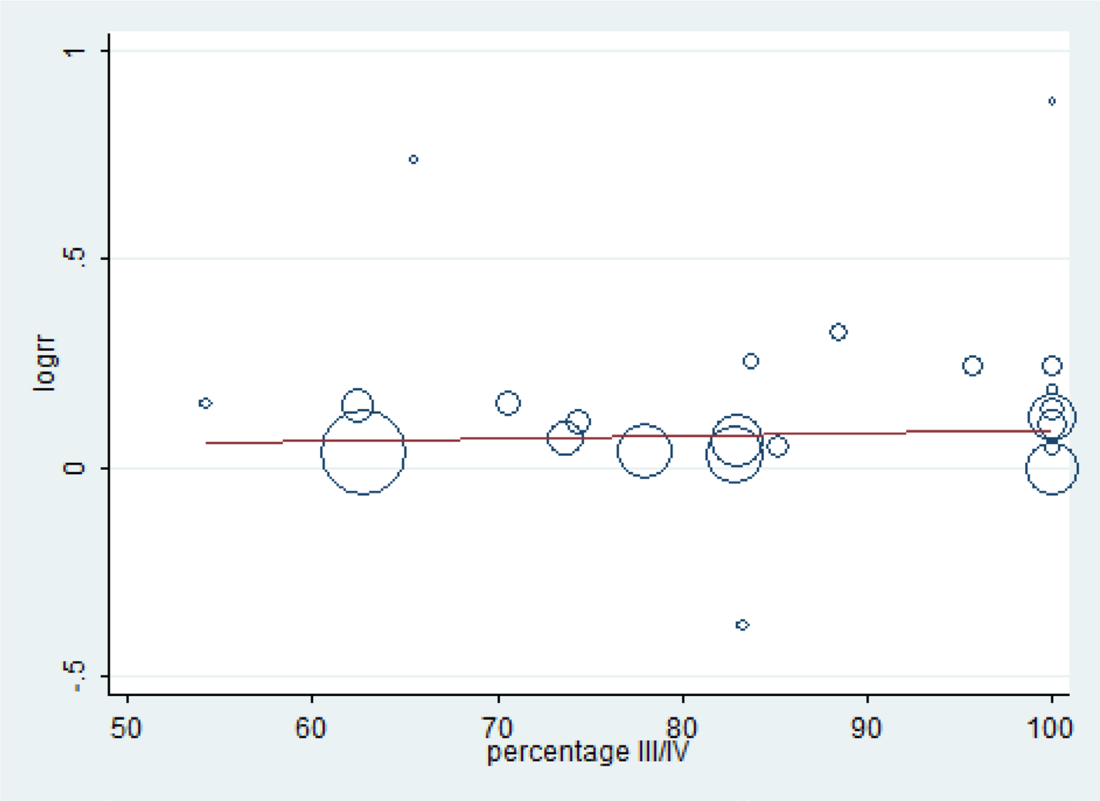

Supplementary Figure 10: Meta-regression for 1-year survival rate based on percentage III/IV (*P* = 0.662).

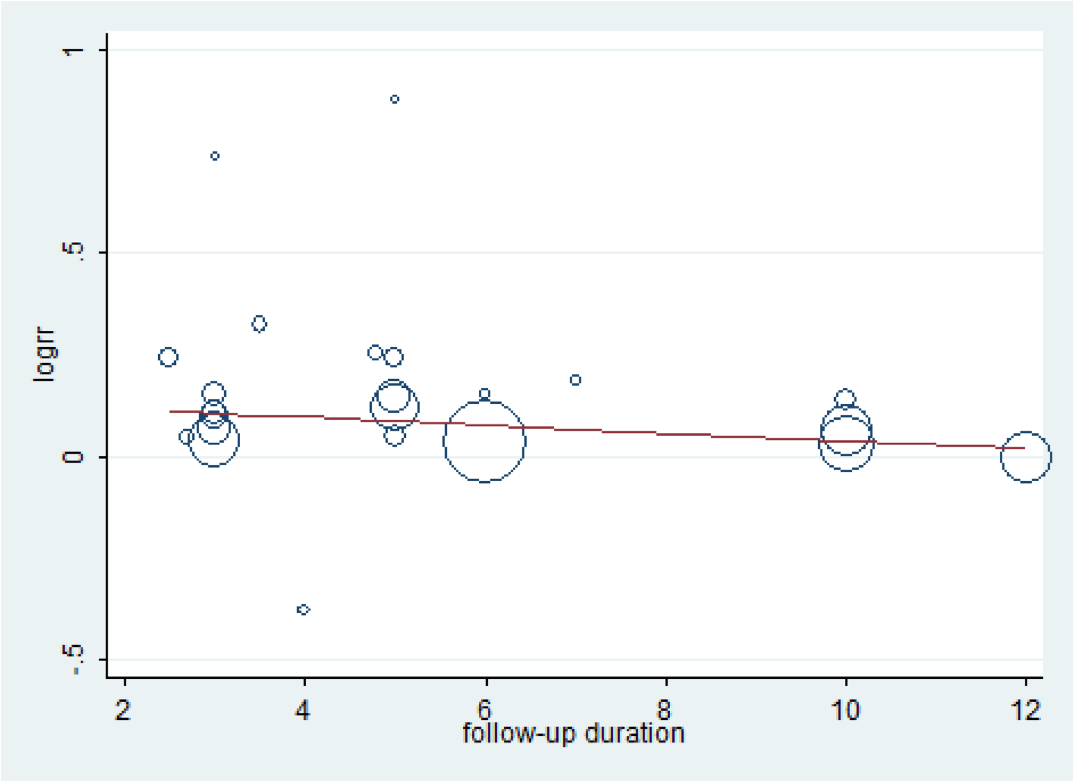

Supplementary Figure 11: Meta-regression for 1-year survival rate based on follow-up duration (*P* = 0.142).

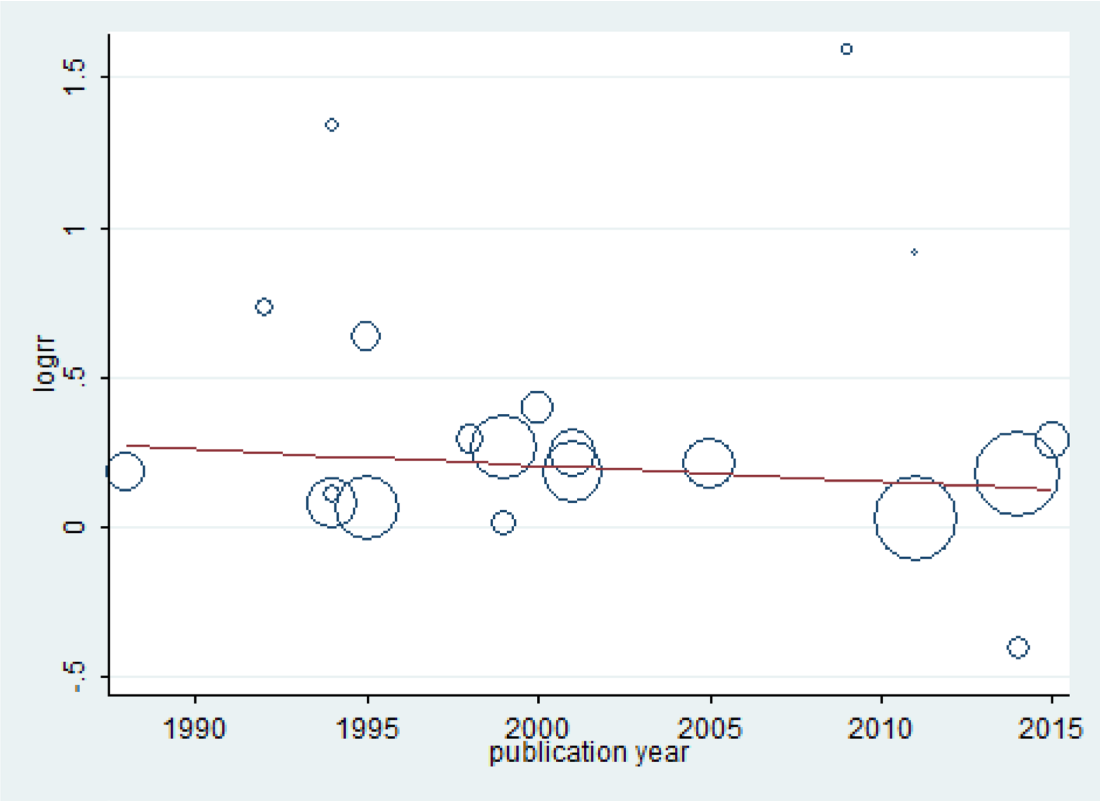

Supplementary Figure 12: Meta-regression for 2-year survival rate based on publication year (*P* = 0.408).

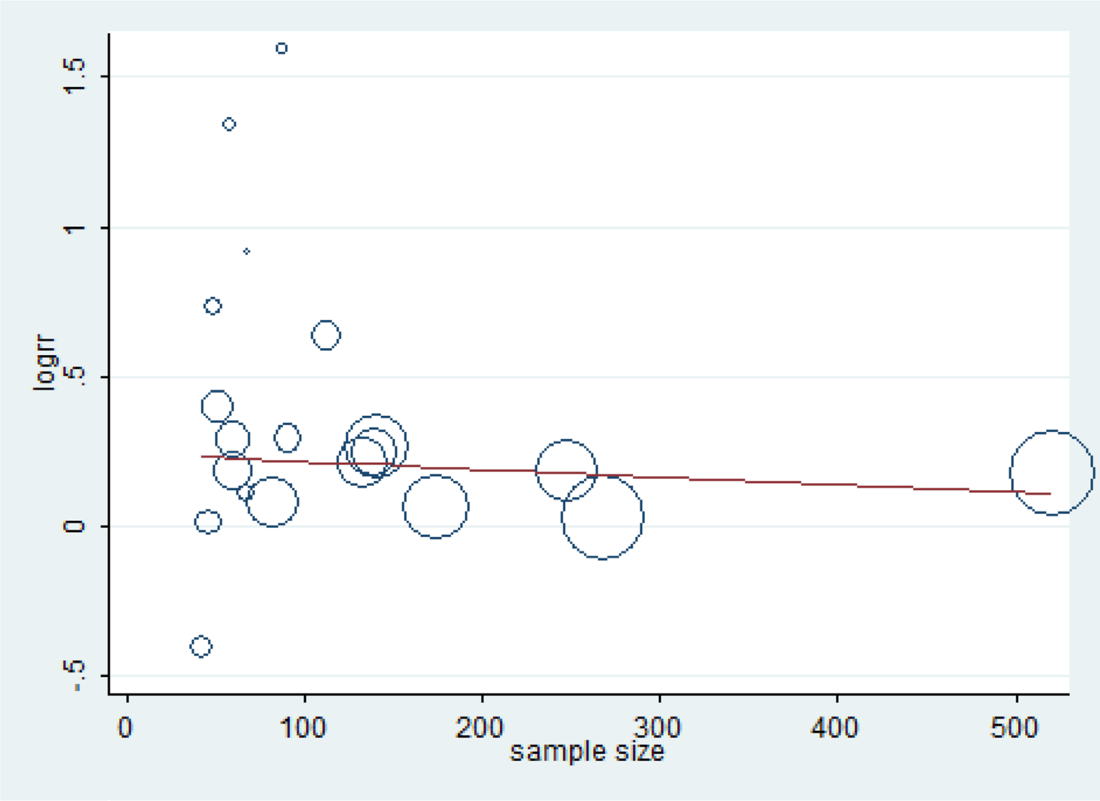

Supplementary Figure 13: Meta-regression for 2-year survival rate based on sample size (*P* = 0.482).

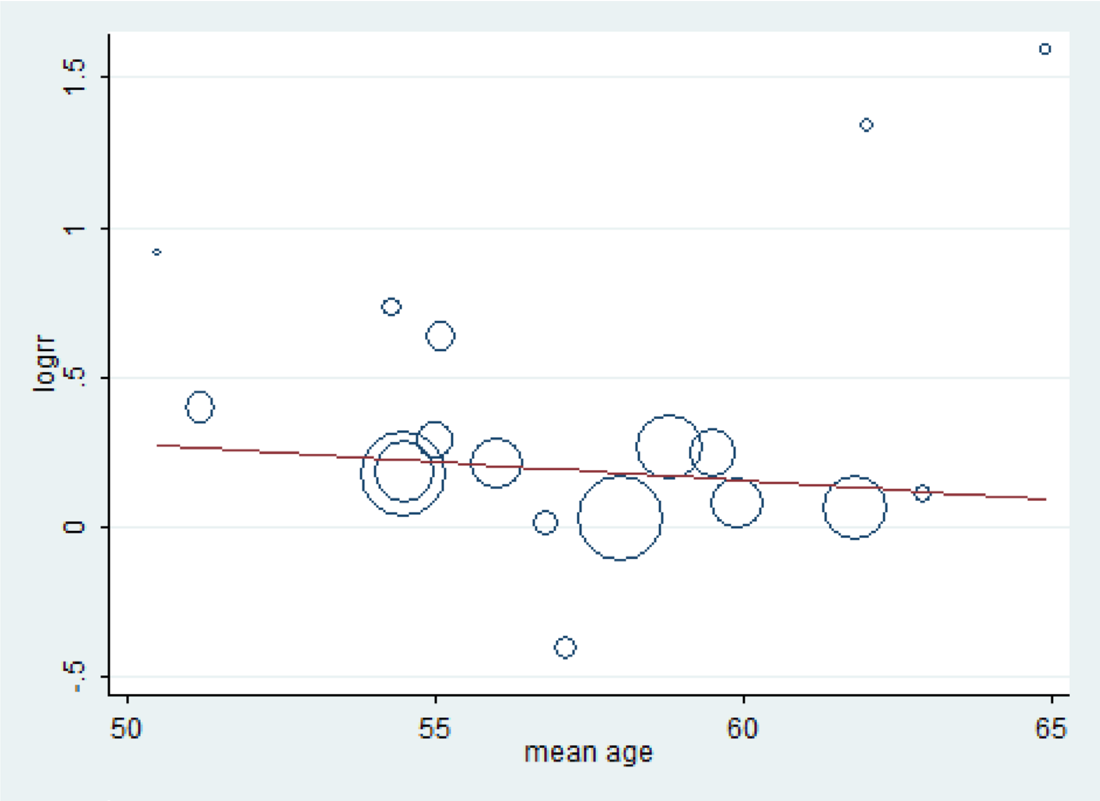

Supplementary Figure 14: Meta-regression for 2-year survival rate based on mean age (*P* = 0.518).

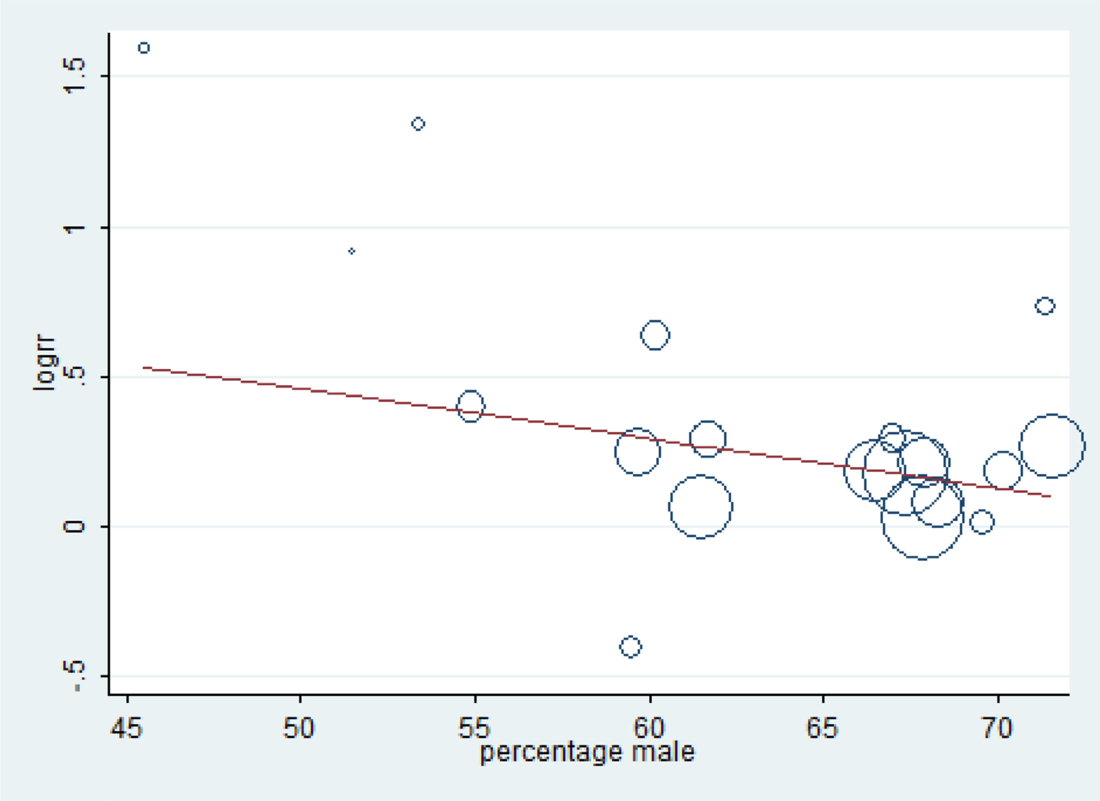

Supplementary Figure 15: Meta-regression for 2-year survival rate based on percentage male (*P* = 0.151).

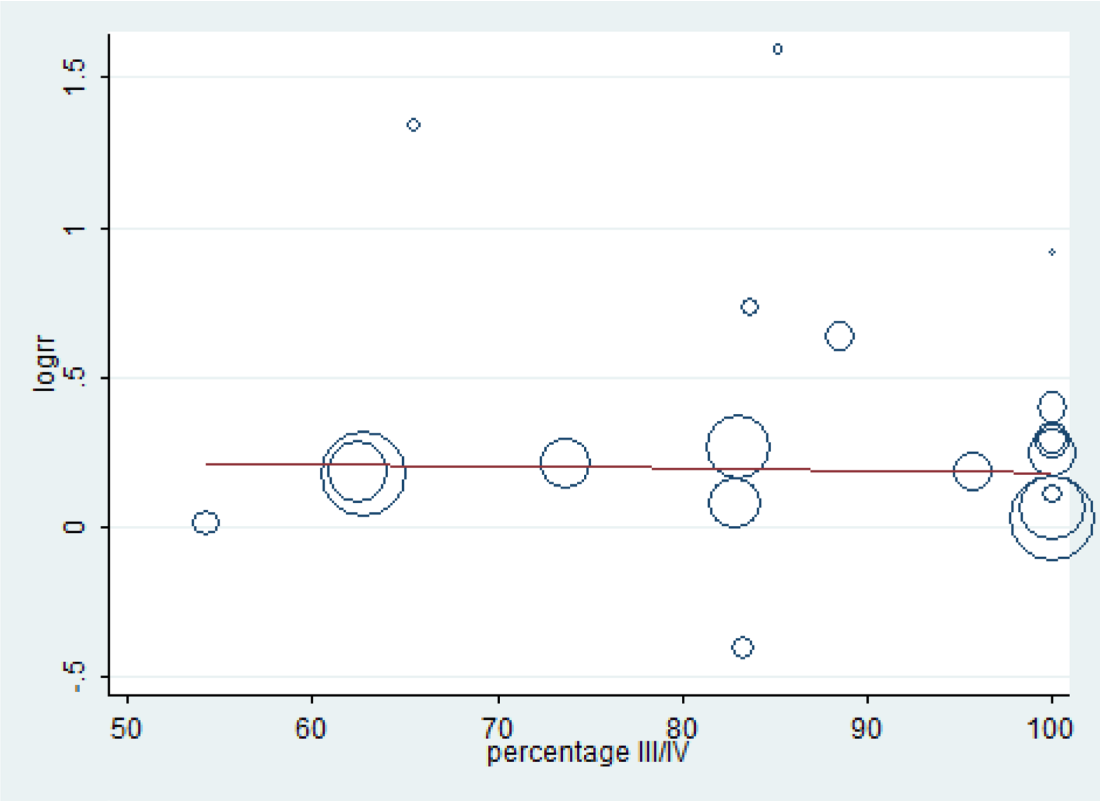

Supplementary Figure 16: Meta-regression for 2-year survival rate based on percentage III/IV (*P* = 0.847).

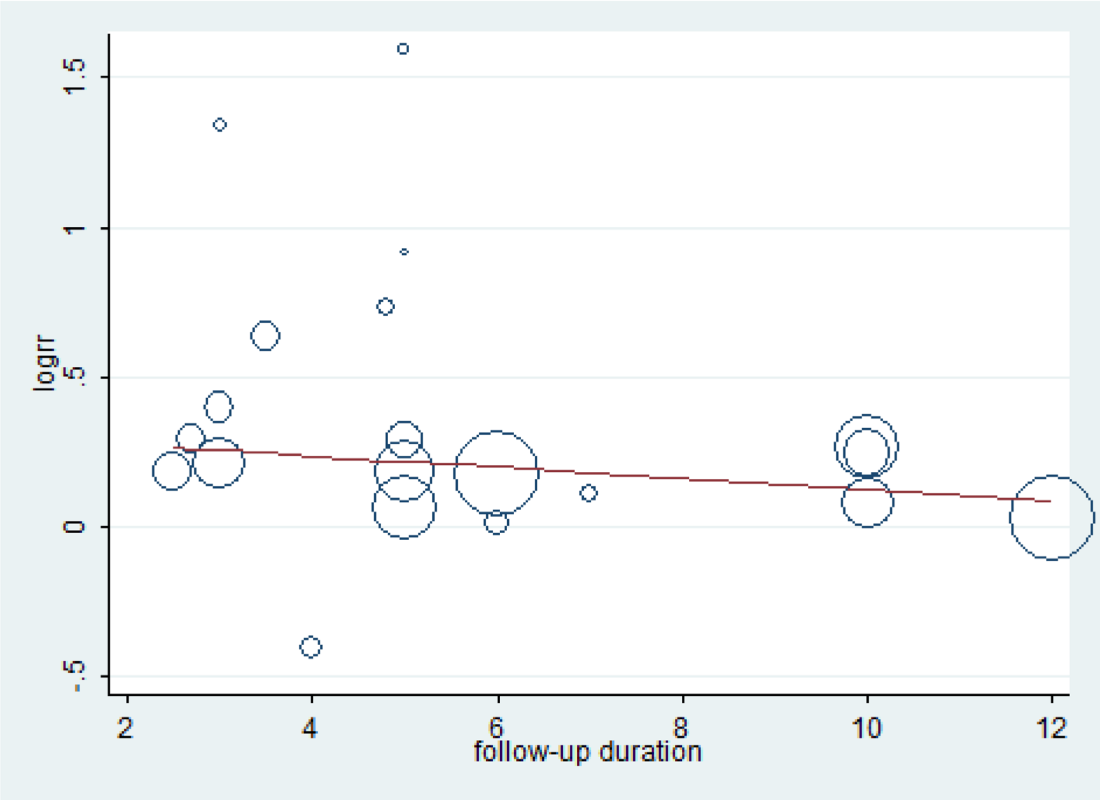

Supplementary Figure 17: Meta-regression for 2-year survival rate based on follow-up duration (*P* = 0.203).

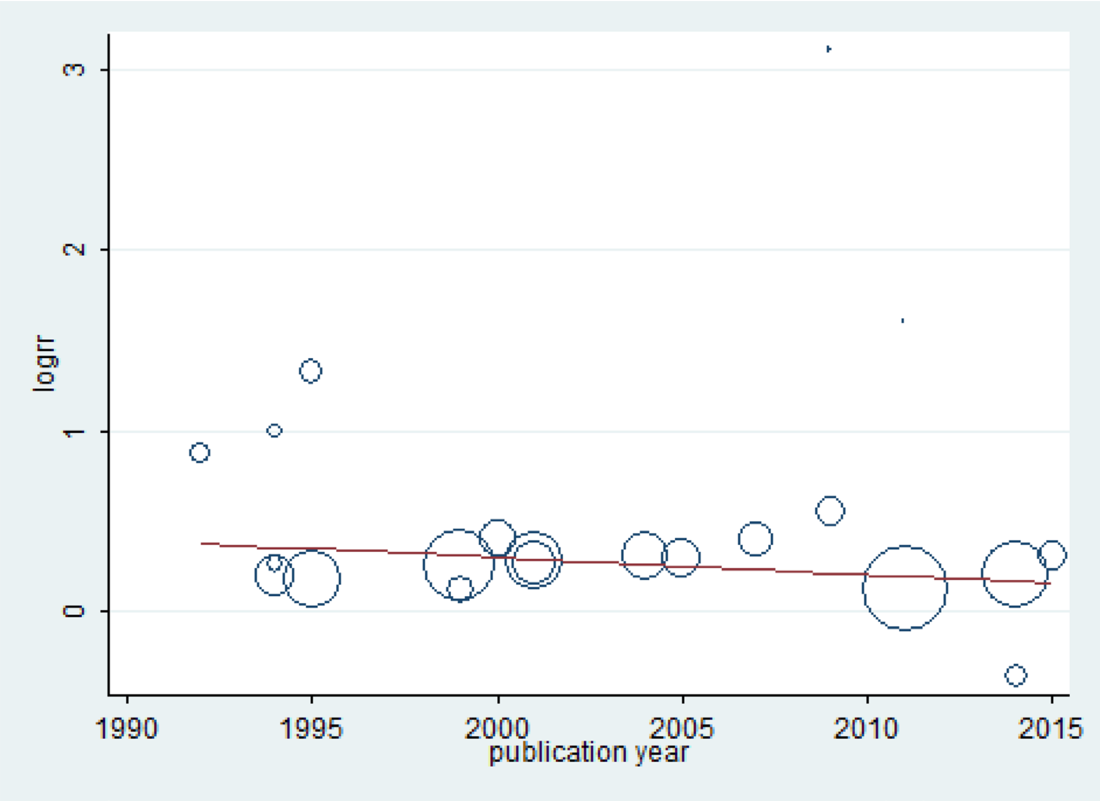

Supplementary Figure 18: Meta-regression for 3-year survival rate based on publication year (*P* = 0.180).

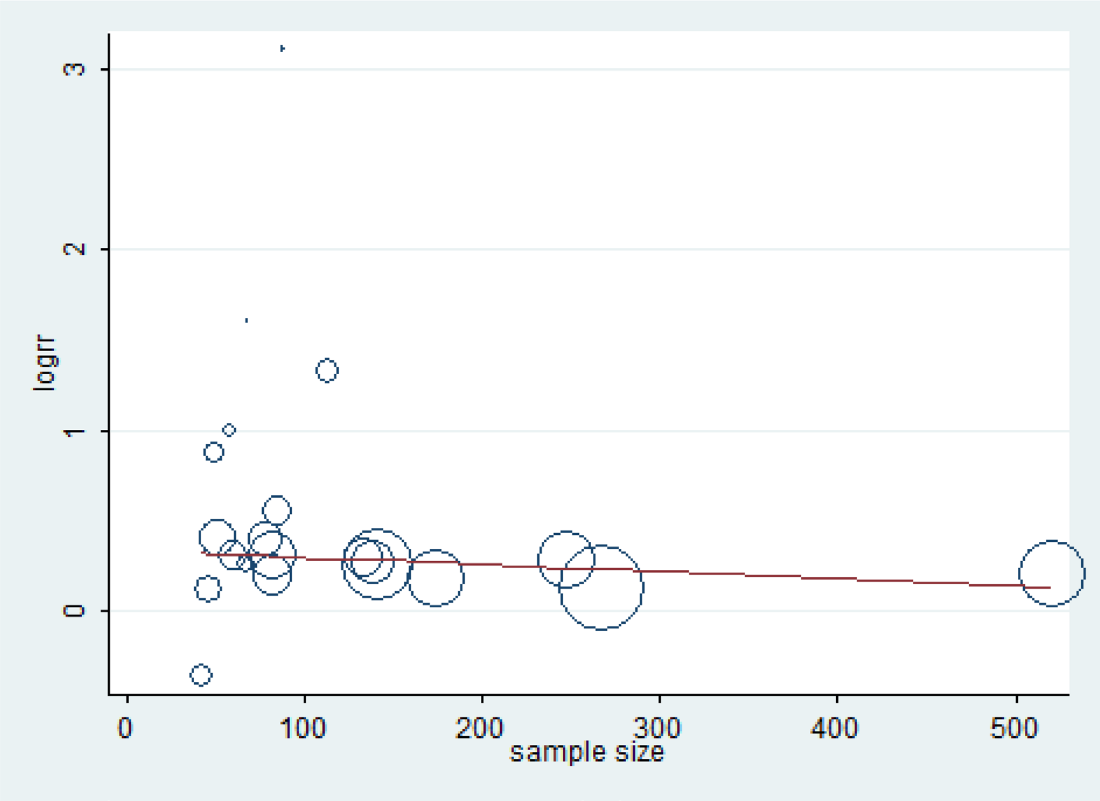

Supplementary Figure 19: Meta-regression for 3-year survival rate based on sample size (*P* = 0.277).

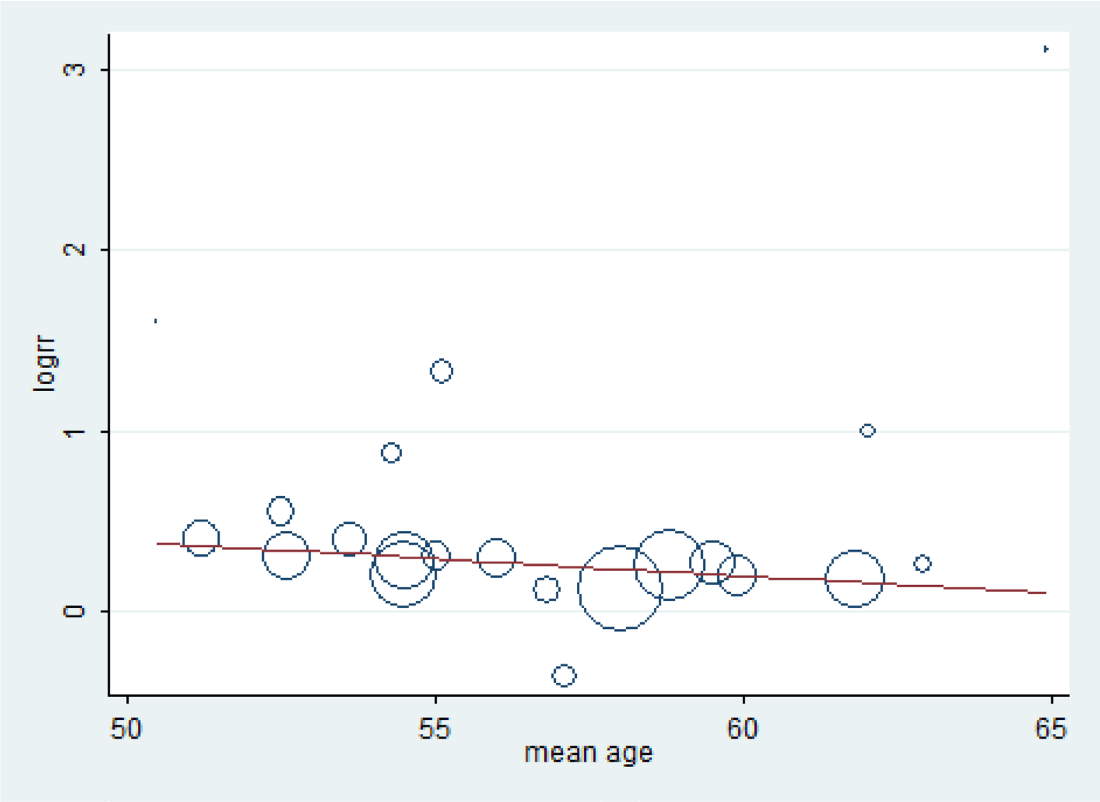

Supplementary Figure 20: Meta-regression for 3-year survival rate based on mean age (*P* = 0.252).

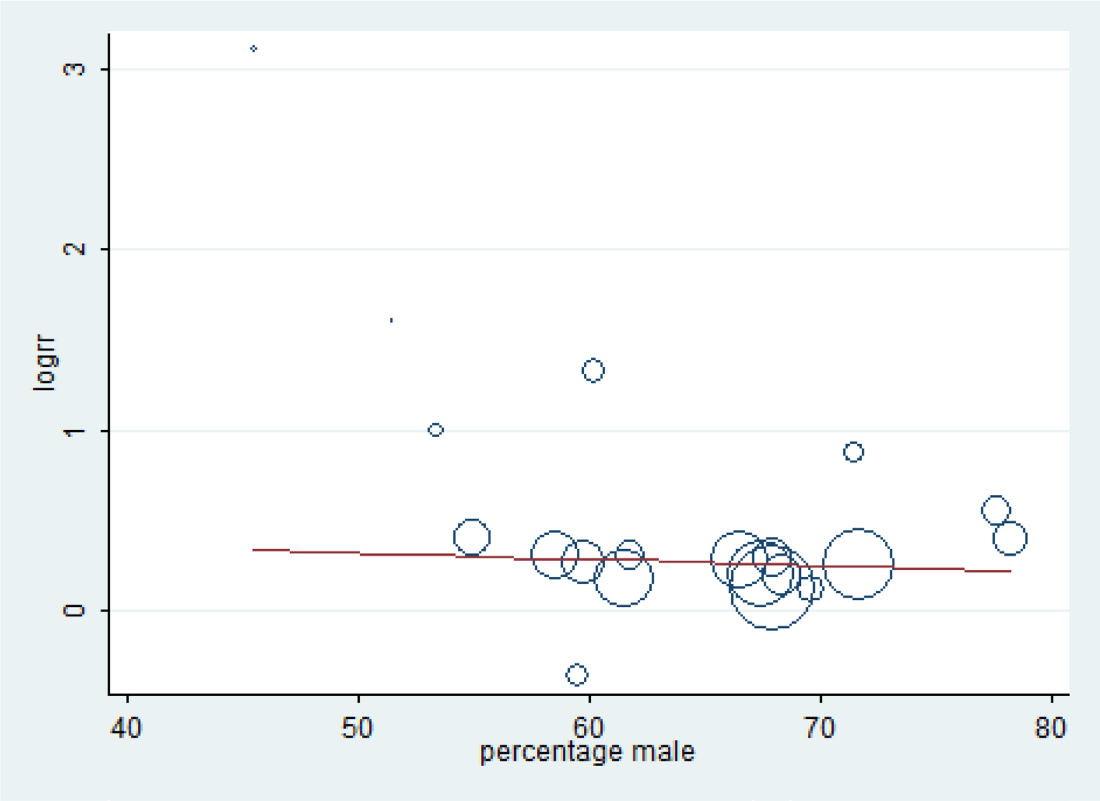

Supplementary Figure 21: Meta-regression for 3-year survival rate based on percentage male (*P* = 0.715).

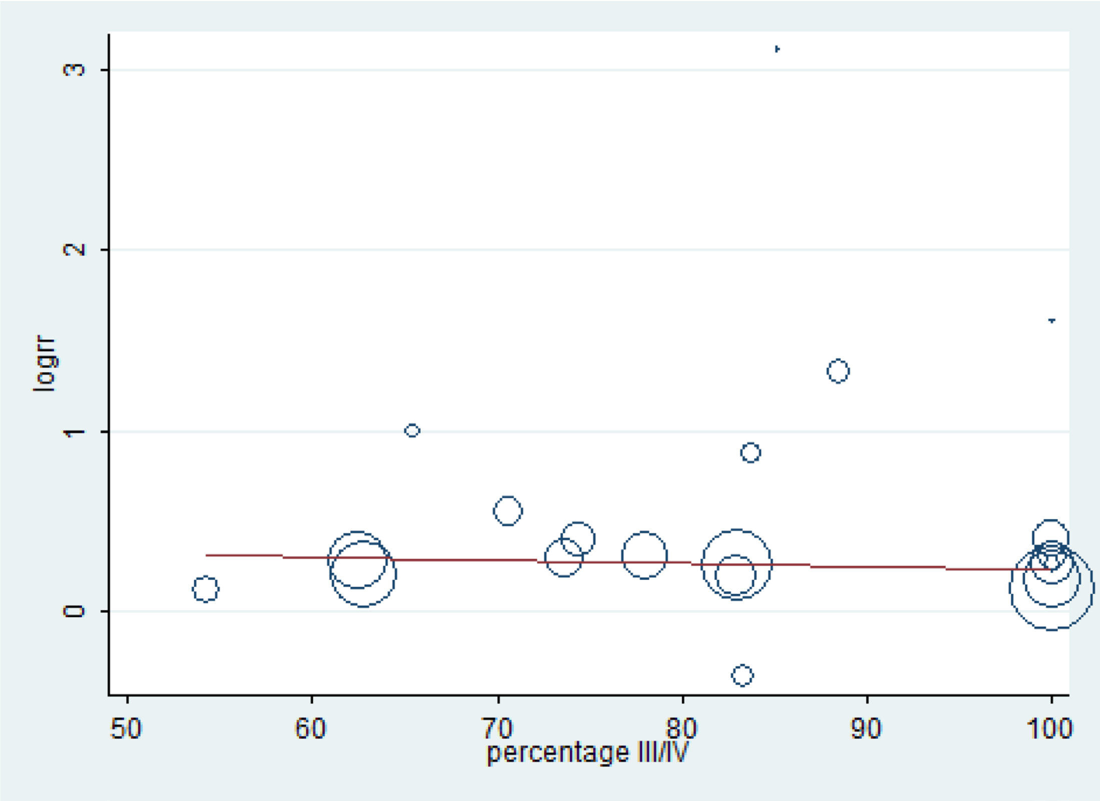

Supplementary Figure 22: Meta-regression for 3-year survival rate based on percentage III/IV (*P* = 0.575).

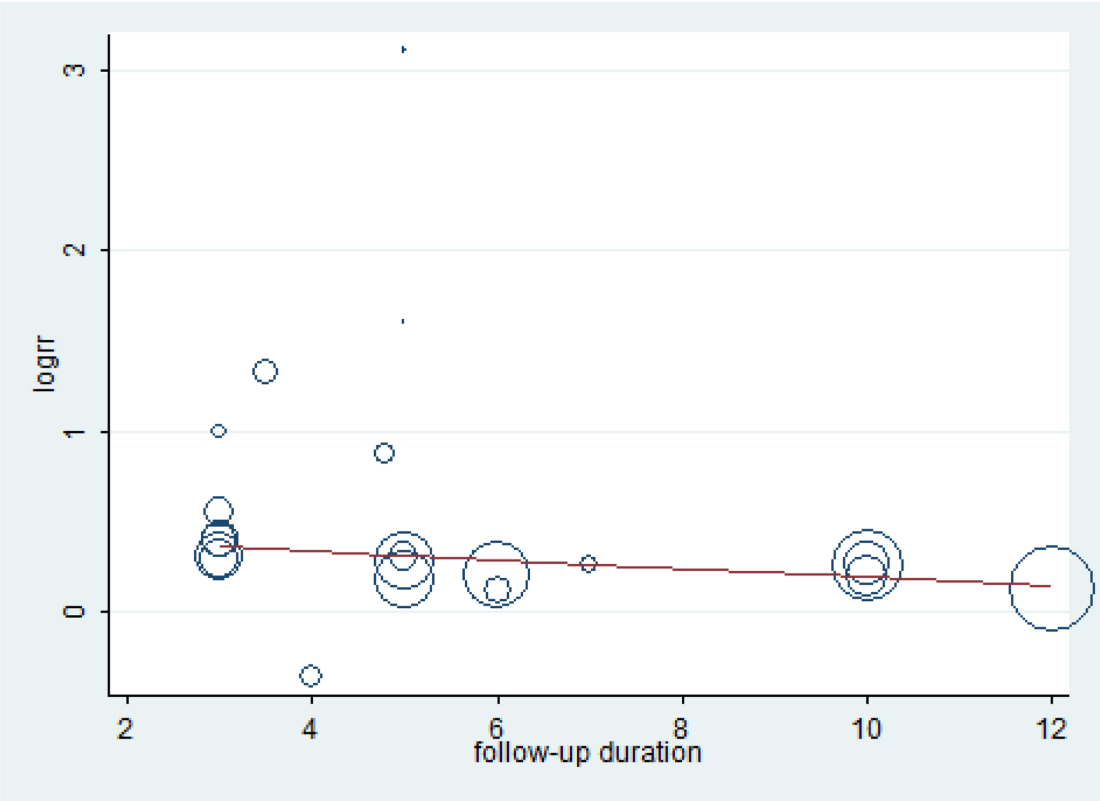

Supplementary Figure 23: Meta-regression for 3-year survival rate based on follow-up duration (*P* = 0.094).

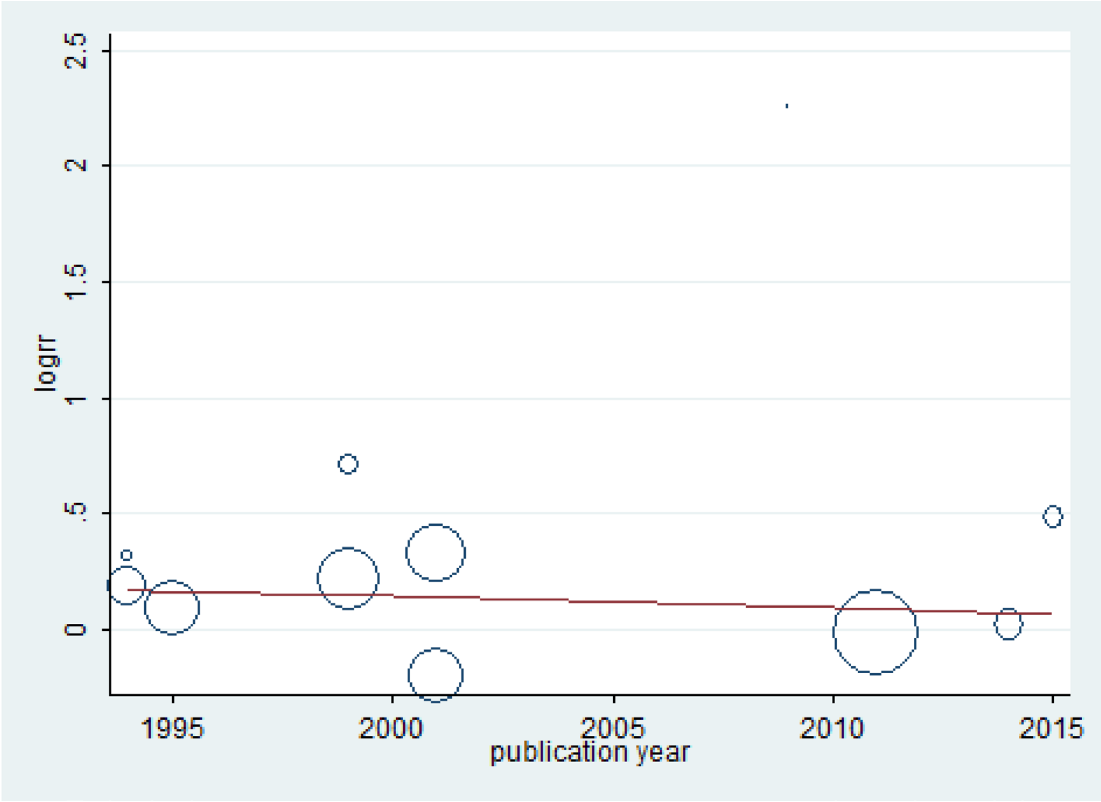

Supplementary Figure 24: Meta-regression for 5-year survival rate based on publication year (*P* = 0.682).

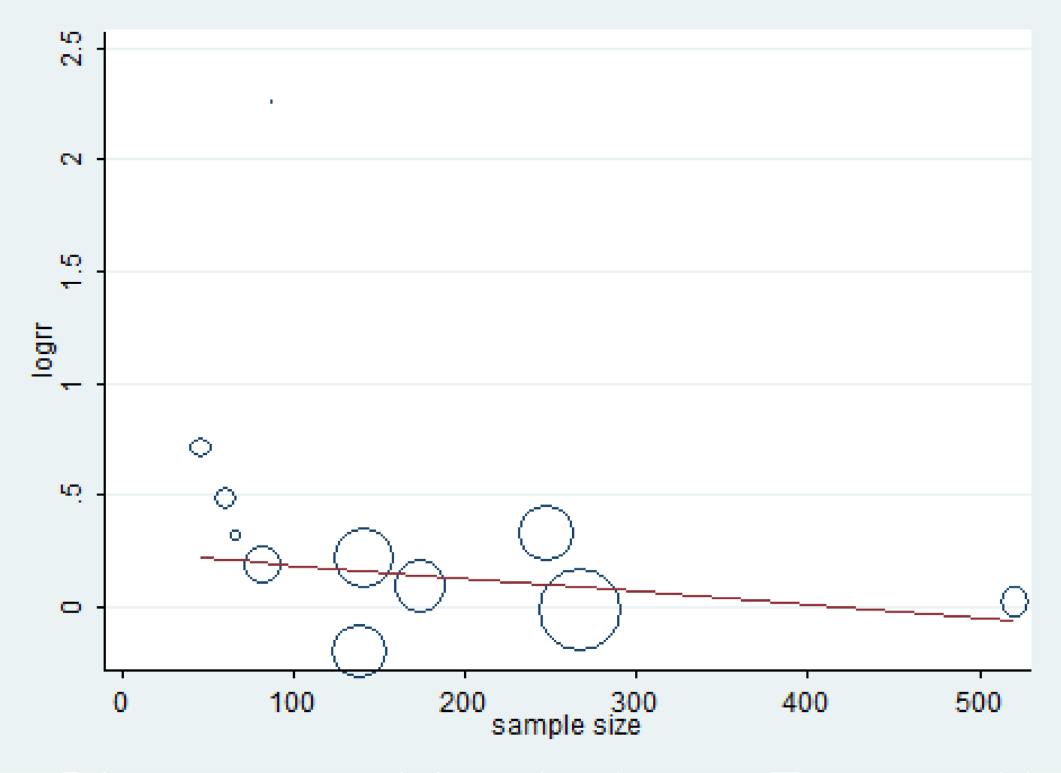

Supplementary Figure 25: Meta-regression for 5-year survival rate based on sample size (*P* = 0.427).

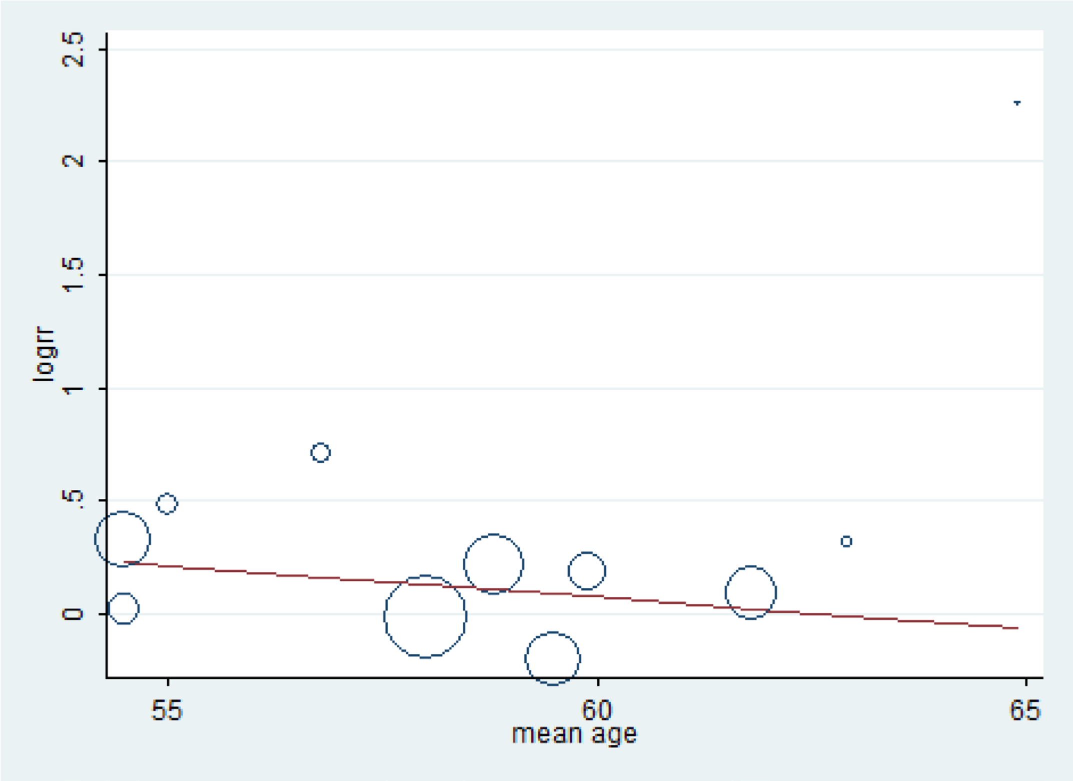

Supplementary Figure 26: Meta-regression for 5-year survival rate based on mean age (*P* = 0.389).

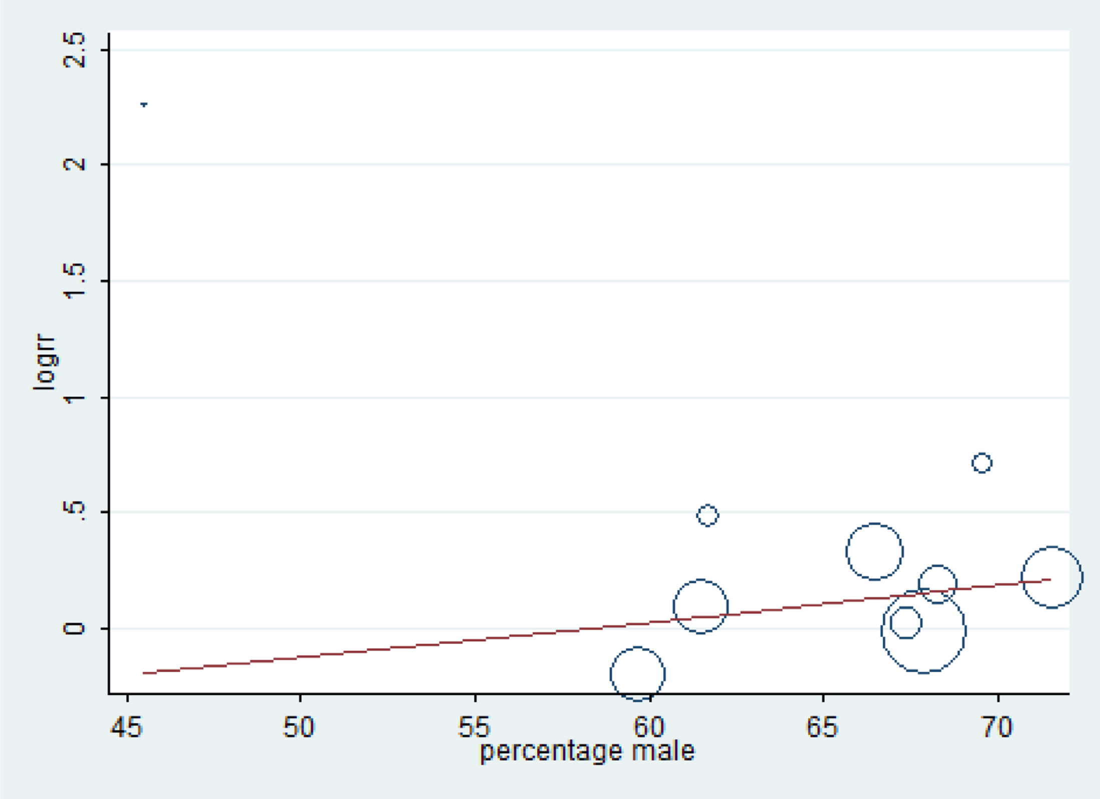

Supplementary Figure 27: Meta-regression for 5-year survival rate based on percentage male (*P* = 0.445).

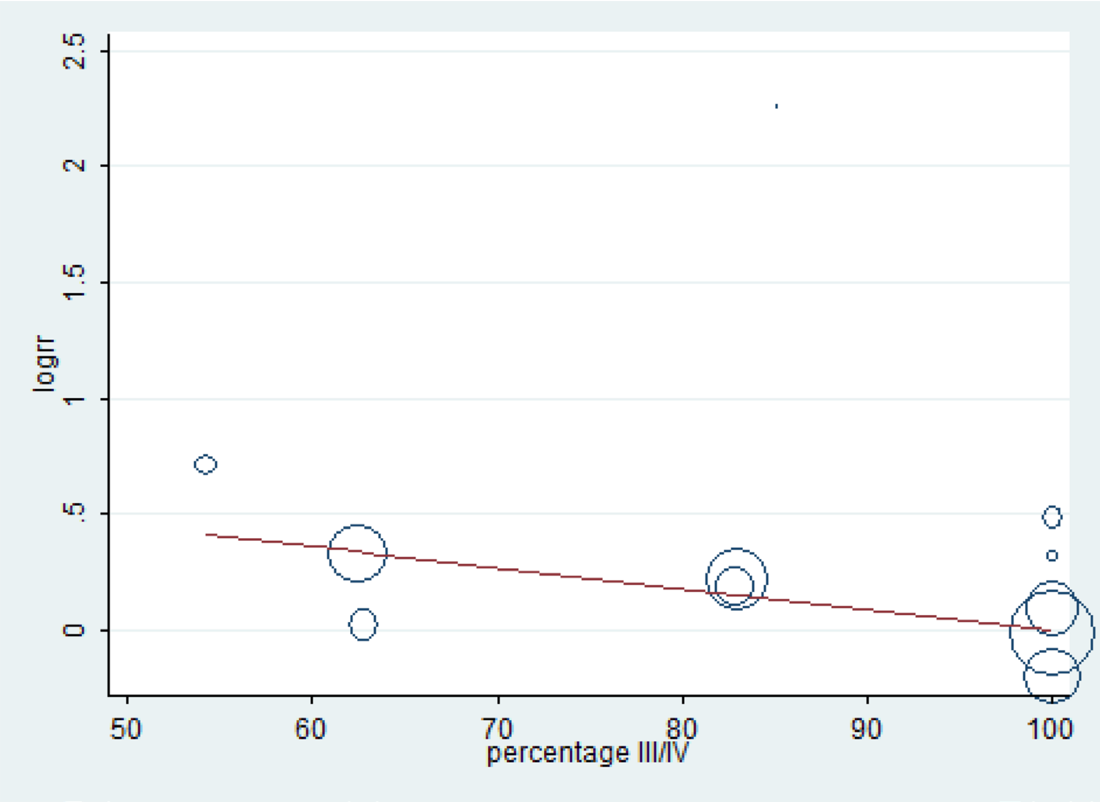

Supplementary Figure 28: Meta-regression for 5-year survival rate based on percentage III/IV (*P* = 0.033).

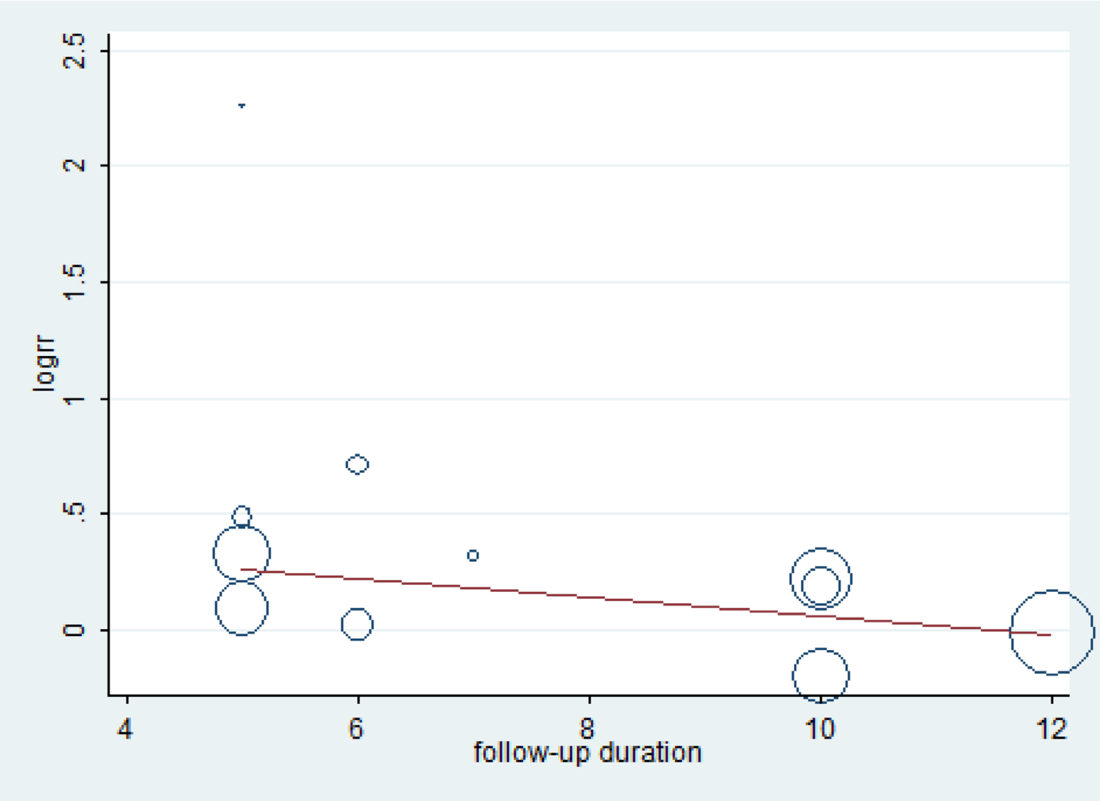

Supplementary Figure 29: Meta-regression for 5-year survival rate based on follow-up duration (*P* = 0.118).

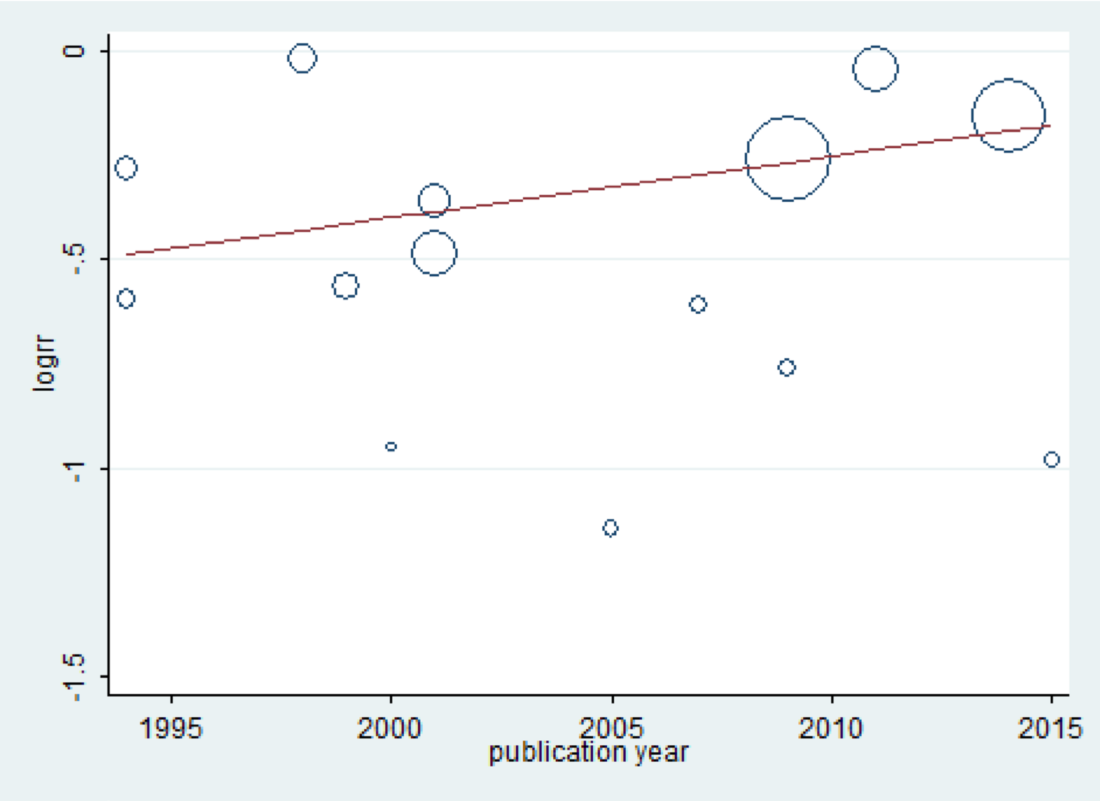

Supplementary Figure 30: Meta-regression for recurrence based on publication year ($P = 0.151$).

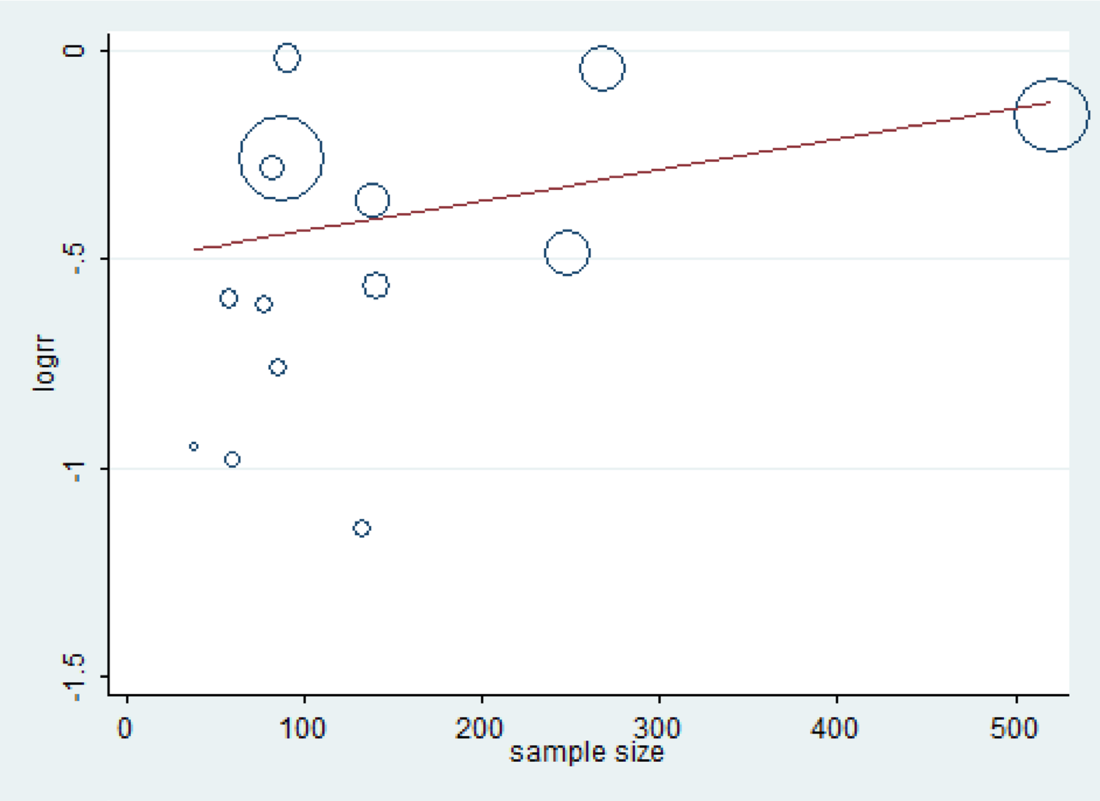

Supplementary Figure 31: Meta-regression for recurrence based on sample size ($P = 0.155$).

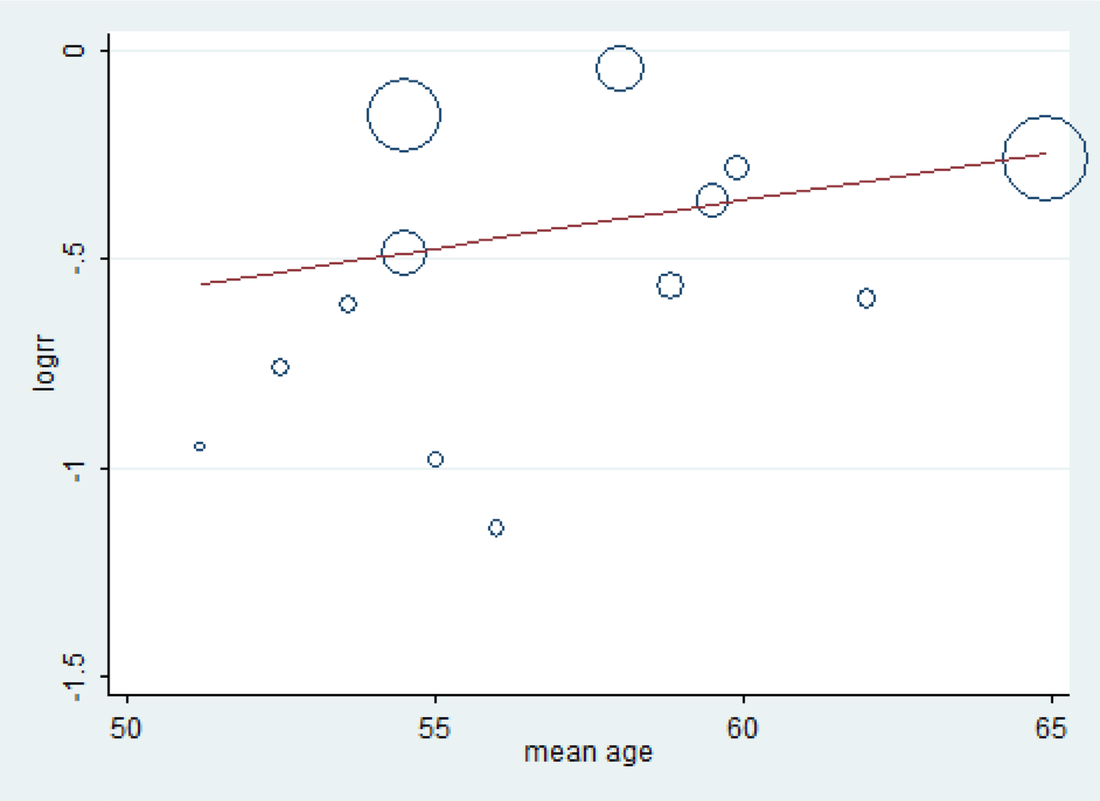

Supplementary Figure 32: Meta-regression for recurrence based on mean age (*P* = 0.316).

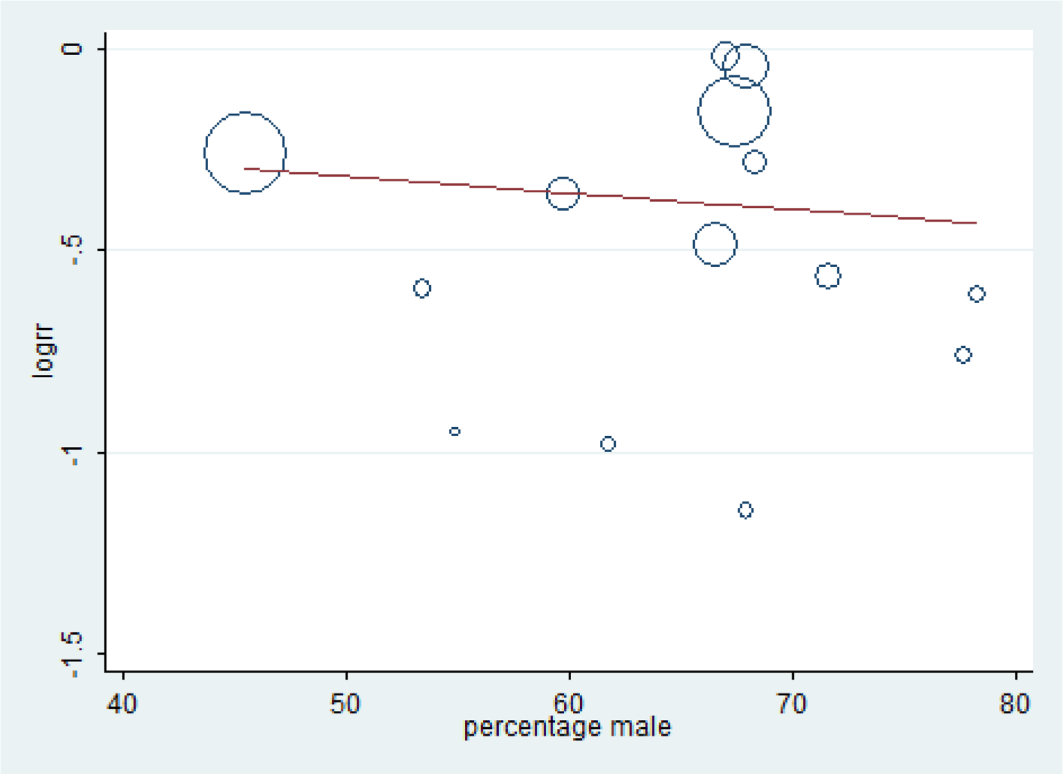

Supplementary Figure 33: Meta-regression for recurrence based on percentage male (*P* = 0.663).

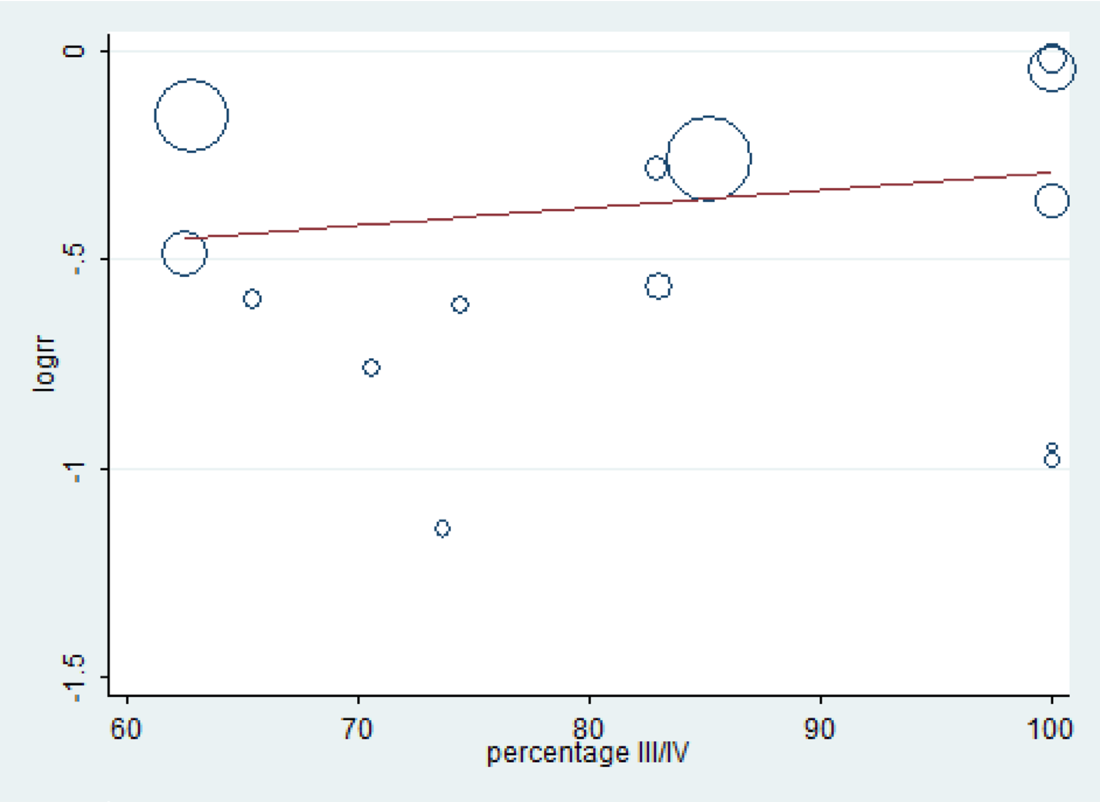

Supplementary Figure 34: Meta-regression for recurrence based on percentage III/IV (*P* = 0.448).

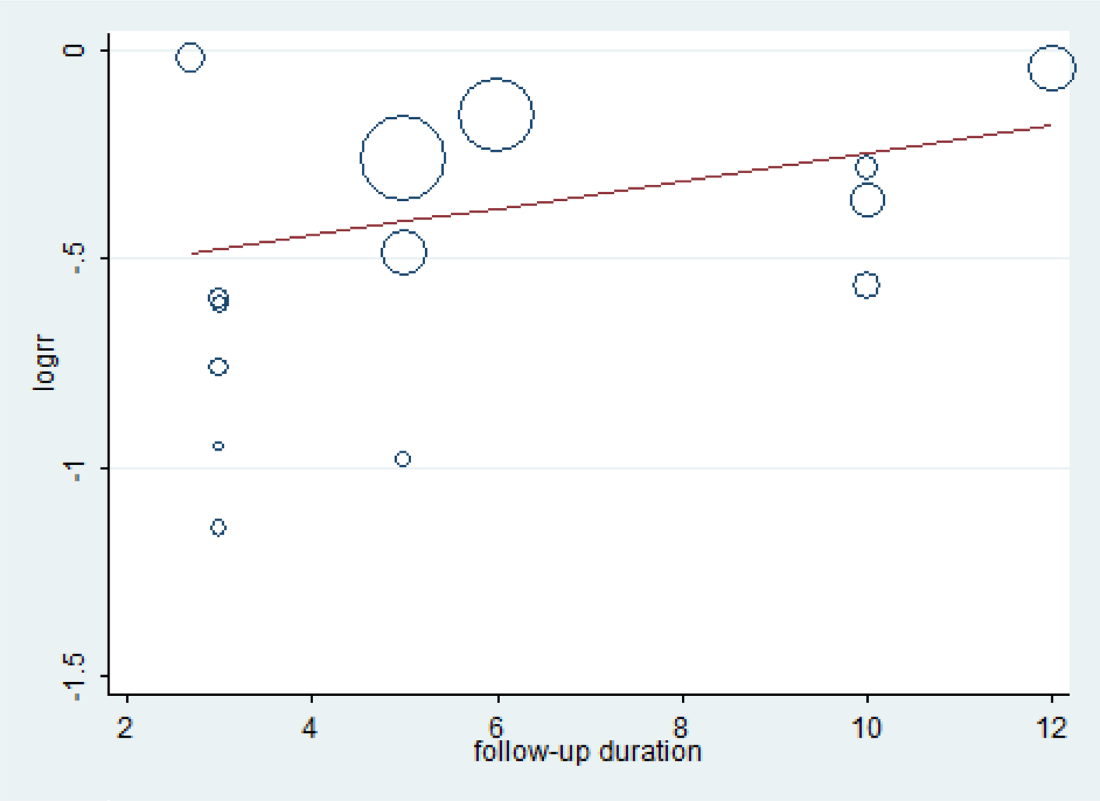

Supplementary Figure 35: Meta-regression for recurrence based on follow-up duration (*P* = 0.203).

1. exp Infusions, Parenteral/
2. Injections, Intraperitoneal/
3. intraperitoneal.mp.
4. intra-peritoneal.mp.
5. peritone*.mp.
6. regional.mp.
7. parenteral.mp.
8. 1 or 2 or 3 or 4 or 5 or 6 or 7
9. exp gastric Neoplasms/
10. exp stomach Neoplasms/
11. (gastr* or stoma* adj5 (cancer* or neoplasm* or malignan* or tumor* or tumour* or carcinom*)).mp
12. 9 or 10 or 11
13. randomized controlled trial.pt.
14. controlled clinical trial.pt.
15. randomized.ab.
16. placebo.ab.
17. drug therapy.fs.
18. randomly.ab.
19. trial.ab.
20. 13 or 14 or 15 or 16 or 17 or 18 or 19
21. 8 and 12 and 20

Supplementary Figure 36: Search strategy in PubMed.

Supplementary Table 1: Sensitivity analysis for 1-year survival rate

Excluding study	RR and 95% CI	*p* value	Heterogeneity (%)	*p* value for heterogeneity
Koga	1.09 (1.04–1.15)	< 0.001	49.6	0.005
Hagiwara	1.10 (1.04–1.15)	< 0.001	50.4	0.004
Hamazoe	1.11 (1.05–1.17)	< 0.001	49.8	0.004
Fujimura	1.09 (1.04–1.14)	< 0.001	41.0	0.024
Sautner	1.10 (1.05–1.15)	< 0.001	51.7	0.003
Takahashi	1.09 (1.04–1.14)	< 0.001	46.6	0.009
Ikeguchi	1.10 (1.04–1.16)	< 0.001	51.9	0.003
Rosen	1.10 (1.05–1.16)	< 0.001	52.8	0.002
Shimoyama	1.10 (1.05–1.15)	< 0.001	52.0	0.002
Fujimoto	1.11 (1.05–1.16)	< 0.001	52.4	0.002
Tan	1.10 (1.05–1.16)	< 0.001	52.4	0.002
Yu	1.10 (1.04–1.15)	< 0.001	50.5	0.004
Yonemura	1.10 (1.05–1.15)	< 0.001	51.7	0.003
Zuo	1.11 (1.05–1.17)	< 0.001	50.7	0.004
Wei	1.10 (1.05–1.16)	< 0.001	52.7	0.002
Ding	1.10 (1.05–1.16)	< 0.001	52.3	0.002
Deng	1.10 (1.04–1.15)	< 0.001	51.4	0.003
Kuramoto	1.10 (1.05–1.16)	< 0.001	52.7	0.002
Miyashiro	1.11 (1.05–1.17)	< 0.001	50.7	0.004
Yang	1.09 (1.04–1.13)	< 0.001	37.2	0.042
Kang	1.11 (1.05–1.17)	< 0.001	51.1	0.003
Huang	1.10 (1.05–1.16)	< 0.001	47.1	0.008
Zheng	1.09 (1.04–1.15)	< 0.001	49.6	0.005

Supplementary Table 2: Sensitivity analysis for 2-year survival rate

Excluding study	RR and 95% CI	*p* value	Heterogeneity (%)	*p* value for heterogeneity
Koga	1.24 (1.12–1.38)	< 0.001	53.6	0.003
Hagiwara	1.22 (1.11–1.34)	< 0.001	49.2	0.008
Hamazoe	1.25 (1.13–1.39)	< 0.001	52.4	0.004
Fujimura	1.21 (1.11–1.32)	< 0.001	41.6	0.030
Sautner	1.24 (1.12–1.37)	< 0.001	53.6	0.003
Takahashi	1.21 (1.10–1.33)	< 0.001	45.5	0.017
Ikeguchi	1.26 (1.13–1.39)	< 0.001	51.5	0.005
Rosen	1.23 (1.12–1.36)	< 0.001	53.1	0.003
Shimoyama	1.25 (1.13–1.38)	< 0.001	53.1	0.003
Fujimoto	1.23 (1.11–1.37)	< 0.001	52.8	0.004
Tan	1.23 (1.11–1.36)	< 0.001	52.0	0.005
Yu	1.24 (1.12–1.38)	< 0.001	53.8	0.003
Yonemura	1.24 (1.12–1.37)	< 0.001	53.3	0.003
Wei	1.24 (1.12–1.38)	< 0.001	53.6	0.003
Kuramoto	1.22 (1.11–1.33)	< 0.001	42.3	0.027
Miyashiro	1.26 (1.14–1.39)	< 0.001	46.4	0.014
Yang	1.23 (1.12–1.36)	< 0.001	52.2	0.004
Kang	1.25 (1.12–1.40)	< 0.001	54.3	0.003
Huang	1.25 (1.14–1.37)	< 0.001	47.5	0.012
Zheng	1.23 (1.11–1.37)	< 0.001	53.1	0.003

Supplementary Table 3: Sensitivity analysis for 3-year survival rate

Excluding study	RR and 95% CI	*p* value	Heterogeneity (%)	*p* value for heterogeneity
Hagiwara	1.32 (1.19–1.47)	< 0.001	41.0	0.030
Hamazoe	1.36 (1.21–1.52)	< 0.001	46.4	0.012
Fujimura	1.33 (1.19–1.48)	< 0.001	41.5	0.028
Sautner	1.35 (1.20–1.51)	< 0.001	46.5	0.012
Takahashi	1.28 (1.18–1.39)	< 0.001	12.2	0.302
Ikeguchi	1.36 (1.21–1.53)	< 0.001	45.8	0.014
Shimoyama	1.35 (1.21–1.52)	< 0.001	46.1	0.013
Fujimoto	1.36 (1.20–1.53)	< 0.001	46.5	0.012
Tan	1.34 (1.19–1.50)	< 0.001	45.8	0.014
Yu	1.35 (1.20–1.52)	< 0.001	46.7	0.012
Yonemura	1.35 (1.20–1.52)	< 0.001	46.6	0.012
Zuo	1.35 (1.20–1.51)	< 0.001	46.5	0.012
Wei	1.35 (1.20–1.51)	< 0.001	46.5	0.012
Ding	1.34 (1.19–1.50)	< 0.001	45.8	0.014
Deng	1.33 (1.19–1.49)	< 0.001	44.0	0.019
Kuramoto	1.33 (1.20–1.47)	< 0.001	37.3	0.048
Miyashiro	1.37 (1.22–1.54)	< 0.001	40.4	0.032
Yang	1.34 (1.20–1.50)	< 0.001	45.0	0.016
Kang	1.36 (1.21–1.54)	< 0.001	47.2	0.011
Huang	1.36 (1.22–1.51)	< 0.001	39.6	0.036
Zheng	1.35 (1.20–1.51)	< 0.001	46.5	0.012

Supplementary Table 4: Sensitivity analysis for 5-year survival rate

Excluding study	RR and 95% CI	*p* value	Heterogeneity (%)	*p* value for heterogeneity
Hamazoe	1.14 (0.97–1.34)	0.123	40.5	0.087
Sautner	1.14 (0.98–1.33)	0.098	40.6	0.087
Ikeguchi	1.15 (0.97–1.37)	0.103	41.3	0.082
Shimoyama	1.11 (0.97–1.28)	0.138	29.4	0.174
Fujimoto	1.13 (0.95–1.34)	0.169	38.1	0.104
Yu	1.09 (0.94–1.27)	0.242	26.7	0.198
Yonemura	1.18 (1.03–1.34)	0.014	12.7	0.326
Kuramoto	1.13 (0.98–1.29)	0.092	29.4	0.175
Miyashiro	1.18 (1.00–1.40)	0.053	31.6	0.156
Kang	1.15 (0.98–1.35)	0.085	41.2	0.083
Zheng	1.12 (0.97–1.30)	0.132	36.1	0.120

Supplementary Table 5: Sensitivity analysis for recurrence

Excluding study	RR and 95% CI	*p* value	Heterogeneity (%)	*p* value for heterogeneity
Hamazoe	0.69 (0.60–0.80)	< 0.001	49.4	0.022
Fujimura	0.70 (0.61–0.81)	< 0.001	47.4	0.029
Rosen	0.68 (0.59–0.79)	< 0.001	46.5	0.033
Fujimoto	0.71 (0.61–0.82)	< 0.001	45.4	0.038
Tan	0.71 (0.61–0.81)	< 0.001	46.2	0.034
Yu	0.71 (0.61–0.83)	< 0.001	43.6	0.047
Yonemura	0.69 (0.60–0.81)	< 0.001	48.9	0.024
Wei	0.73 (0.64–0.83)	< 0.001	33.7	0.113
Ding	0.70 (0.61–0.81)	< 0.001	47.1	0.031
Deng	0.71 (0.62–0.82)	< 0.001	44.2	0.043
Kuramoto	0.67 (0.57–0.80)	< 0.001	48.0	0.027
Miyashiro	0.67 (0.58–0.78)	< 0.001	43.2	0.049
Kang	0.67 (0.57–0.79)	< 0.001	45.0	0.039
Zheng	0.72 (0.63–0.82)	< 0.001	41.2	0.060

Supplementary Table 6: Subgroup analyses for survival rate at different follow-up durations and recurrence. See Supplementary_Table_6
