# Supplementary material for: Efficacy and safety of intraperitoneal chemotherapy in patients with advanced gastric cancer: a cumulative meta-analysis of randomized controlled trials
Source: Oncotarget. 2017 Sep 11;8(46):81125–36. doi: 10.18632/oncotarget.20818 (PMC5655267; doi:10.18632/oncotarget.20818)
Supplement: Supplementary file 2 [file oncotarget-08-81125-s002.docx]

**Supplementary Table 6: Subgroup analyses for survival rate at different follow-up durations and recurrence**

| Outcomes | Group | RR and 95%CI | | P value | Heterogeneity (%) | | | P value for heterogeneity | P value for heterogeneity between subgroups |
| --- | --- | --- | --- | --- | --- | --- | --- | --- | --- |
| 1-year survival rate | **Publication year** | | | | | | | | |
|  | 2000 or after | 1.08 (1.02-1.14) | | 0.014 | 49.6 | | | 0.021 | 1.000 |
|  | Before 2000 | 1.15 (1.05-1.27) | | 0.003 | 59.2 | | | 0.009 |  |
|  | **Country** | | | | | | | | |
|  | Japan | 1.11 (1.03-1.20) | | 0.004 | 55.1 | | | 0.014 | 1.000 |
|  | Korea | 1.07 (0.96-1.20) | | 0.220 | 57.9 | | | 0.123 |  |
|  | China | 1.11 (0.99-1.24) | | 0.081 | 64.7 | | | 0.006 |  |
|  | Austria | 1.10 (0.86-1.39) | | 0.449 | 0.0 | | | 0.586 |  |
|  | **Sample size** | | | | | | | | |
|  | ≥ 100 | 1.07 (1.02-1.12) | | 0.004 | 30.6 | | | 0.184 | 1.000 |
|  | < 100 | 1.14 (1.04-1.25) | | 0.007 | 63.0 | | | 0.001 |  |
|  | **Mean age** | | | | | | | | |
|  | ≥ 60 years | 1.19 (0.98-1.45) | | 0.086 | 58.3 | | | 0.066 | 0.161 |
|  | < 60 years | 1.08 (1.03-1.14) | | 0.002 | 50.1 | | | 0.010 |  |
|  | **Percentage male** | | | | | | | | |
|  | ≥ 70% | 1.11 (1.04-1.19) | | 0.003 | 0.0 | | | 0.434 | 0.356 |
|  | < 70% | 1.09 (1.03-1.16) | | 0.004 | 58.1 | | | 0.001 |  |
|  | **Percentage III/IV gastric cancer** | | | | | | | | |
|  | ≥ 90% | 1.14 (1.04-1.26) | | 0.006 | 52.7 | | | 0.031 | 0.157 |
|  | < 90% | 1.08 (1.02-1.14) | | 0.006 | 48.3 | | | 0.022 |  |
|  | **Follow-up duration (years)** | | | | | | | | |
|  | ≥ 5 years | 1.08 (1.02-1.14) | | 0.005 | 45.9 | | | 0.041 | 0.291 |
|  | < 5 years | 1.13 (1.02-1.24) | | 0.014 | 55.8 | | | 0.012 |  |
| 2-year survival rate | **Publication year** | | | | | | | | |
|  | 2000 or after | 1.20 (1.06-1.36) | | 0.005 | 51.0 | | | 0.031 | 1.000 |
|  | Before 2000 | 1.29 (1.10-1.53) | | 0.002 | 55.9 | | | 0.015 |  |
|  | **Country** | | | | | | | | |
|  | Japan | 1.30 (1.10-1.53) | | 0.002 | 69.9 | | | <0.001 | 1.000 |
|  | Korea | 1.19 (1.06-1.34) | | 0.003 | 0.0 | | | 0.956 |  |
|  | China | 1.21 (0.95-1.56) | | 0.127 | 44.2 | | | 0.127 |  |
|  | Austria | 1.26 (0.89-1.79) | | 0.192 | 0.0 | | | 0.635 |  |
|  | **Sample size** | | | | | | | | |
|  | ≥ 100 | 1.19 (1.09-1.31) | | <0.001 | 40.2 | | | 0.110 | 1.000 |
|  | < 100 | 1.34 (1.07-1.68) | | 0.010 | 61.2 | | | 0.003 |  |
|  | **Mean age** | | | | | | | | |
|  | ≥ 60 years | 1.96 (0.87-4.39) | | 0.103 | 85.6 | | | <0.001 | 1.000 |
|  | < 60 years | 1.21 (1.10-1.33) | | <0.001 | 40.8 | | | 0.056 |  |
|  | **Percentage male** | | | | | | | | |
|  | ≥ 70% | 1.32 (1.09-1.61) | | 0.005 | 20.9 | | | 0.283 | 0.503 |
|  | < 70% | 1.23 (1.09-1.37) | | <0.001 | 56.9 | | | 0.003 |  |
|  | **Percentage III/IV gastric cancer** | | | | | | | | |
|  | ≥ 90% | 1.14 (1.04-1.24) | | 0.005 | 0.0 | | | 0.466 | 0.164 |
|  | < 90% | 1.29 (1.10-1.52) | | 0.002 | 65.7 | | | 0.001 |  |
|  | **Follow-up duration (years)** | | | | | | | | |
|  | ≥ 5 years | 1.17 (1.07-1.27) | | <0.001 | 28.1 | | | 0.169 | 0.045 |
|  | < 5 years | 1.41 (1.10-1.80) | | 0.006 | 64.0 | | | 0.007 |  |
| 3-year survival rate | **Publication year** | | | | | | | | |
|  | 2000 or after | 1.28 (1.15-1.42) | | <0.001 | 19.0 | | | 0.251 | 1.000 |
|  | Before 2000 | 1.53 (1.17-1.98) | | 0.002 | 66.5 | | | 0.004 |  |
|  | **Country** | | | | | | | | |
|  | Japan | 1.44 (1.17-1.79) | | 0.001 | | 70.1 | | <0.001 | 1.000 |
|  | Korea | 1.26 (1.08-1.48) | | 0.004 | | 0.0 | | 0.648 |  |
|  | China | 1.36 (1.17-1.59) | | <0.001 | | 0.0 | | 0.433 |  |
|  | Austria | 1.29 (0.58-2.86) | | 0.534 | | - | | - |  |
|  | **Sample size** | | | | | | | | |
|  | ≥ 100 | 1.30 (1.14-1.49) | | <0.001 | | 56.5 | | 0.024 | 0.281 |
|  | < 100 | 1.41 (1.17-1.69) | | <0.001 | | 34.2 | | 0.109 |  |
|  | **Mean age** | | | | | | | | |
|  | ≥ 60 years | 1.81 (0.85-3.85) | | 0.124 | | 70.8 | | 0.016 | 1.000 |
|  | < 60 years | 1.33 (1.20-1.49) | | <0.001 | | 40.8 | | 0.041 |  |
|  | **Percentage male** | | | | | | | | |
|  | ≥ 70% | 1.49 (1.18-1.87) | | 0.001 | | 30.3 | | 0.230 | 0.596 |
|  | < 70% | 1.31 (1.15-1.50) | | <0.001 | | 50.2 | | 0.011 |  |
|  | **Percentage III/IV gastric cancer** | | | | | | | | |
|  | ≥ 90% | 1.21 (1.08-1.35) | | 0.001 | | 0.0 | | 0.780 | 0.140 |
|  | < 90% | 1.42 (1.21-1.67) | | <0.001 | | 56.8 | | 0.005 |  |
|  | **Follow-up duration (years)** | | | | | | | | |
|  | ≥ 5 years | 1.23 (1.13-1.33) | | <0.001 | | 0.0 | | 0.732 | 0.012 |
|  | < 5 years | 1.60 (1.24-2.08) | | <0.001 | | 62.7 | | 0.006 |  |
| 5-year survival rate | **Publication year** | | | | | | | | |
|  | 2000 or after | 1.09 (0.86-1.40) | | 0.475 | | 56.0 | | 0.044 | 0.199 |
|  | Before 2000 | 1.22 (1.02-1.45) | | 0.028 | | 0.0 | | 0.697 |  |
|  | **Country** | | | | | | | | |
|  | Japan | 1.09 (0.91-1.30) | | 0.361 | | 44.2 | | 0.096 | 0.325 |
|  | Korea | 1.29 (1.00-1.68) | | 0.053 | | 4.2 | | 0.307 |  |
|  | China | 1.63 (0.79-3.34) | | 0.187 | | - | | - |  |
|  | Austria | 1.37 (0.33-5.67) | | 0.661 | | - | | - |  |
|  | **Sample size** | | | | | | | | |
|  | ≥ 100 | 1.08 (0.92-1.26) | | 0.339 | | 41.9 | | 0.126 | 0.086 |
|  | < 100 | 1.44 (1.05-1.98) | | 0.025 | | 0.0 | | 0.446 |  |
|  | **Mean age** | | | | | | | | |
|  | ≥ 60 years | 1.30 (0.64-1.64) | | 0.469 | | 25.7 | | 0.260 | 1.000 |
|  | < 60 years | 1.14 (0.96-1.35) | | 0.130 | | 44.7 | | 0.081 |  |
|  | **Percentage male** | | | | | | | | |
|  | ≥ 70% | 1.25 (0.95-1.63) | | 0.109 | | - | | - | 0.653 |
|  | < 70% | 1.13 (0.94-1.35) | | 0.185 | | 44.5 | | 0.072 |  |
|  | **Percentage III/IV gastric cancer** | | | | | | | | |
|  | ≥ 90% | 0.99 (0.85-1.14) | | 0.870 | | 4.8 | | 0.380 | 0.010 |
|  | < 90% | 1.30 (1.10-1.53) | | 0.002 | | 0.0 | | 0.482 |  |
|  | **Follow-up duration (years)** | | | | | | | | |
|  | ≥ 5 years | 1.14 (0.98-1.32) | | 0.086 | | 34.5 | | 0.123 | - |
|  | < 5 years | - | | - | | - | | - |  |
| Recurrence | **Publication year** | | | | | | | | |
|  | 2000 or after | 0.69 (0.58-0.81) | | <0.001 | | 55.0 | | 0.018 | 0.701 |
|  | Before 2000 | 0.72 (0.54-0.94) | | 0.018 | | 14.6 | | 0.319 |  |
|  | **Country** | | | | | | | | |
|  | Japan | 0.77 (0.67-0.87) | | <0.001 | | 4.6 | | 0.387 | 0.004 |
|  | Korea | 0.74 (0.54-1.02) | | 0.063 | | 76.3 | | 0.048 |  |
|  | China | 0.42 (0.30-0.59) | | <0.001 | | 0.0 | | 0.871 |  |
|  | Austria | 0.98 (0.63-1.52) | | 0.923 | | - | | - |  |
|  | **Sample size** | | | | | | | | |
|  | ≥ 100 | 0.70 (0.56-0.88) | | 0.002 | | 63.6 | | 0.017 | 1.000 |
|  | < 100 | 0.66 (0.52-0.84) | | 0.001 | | 41.7 | | 0.101 |  |
|  | **Mean age** | | | | | | | | |
|  | ≥ 60 years | 0.72 (0.52-1.01) | 0.054 | | 31.1 | | 0.228 | | 0.497 |
|  | < 60 years | 0.65 (0.54-0.79) | <0.001 | | 51.9 | | 0.022 | |  |
|  | **Percentage male** | | | | | | | | |
|  | ≥ 70% | 0.54 (0.38-0.75) | <0.001 | | 0.0 | | 0.893 | | 0.030 |
|  | < 70% | 0.73 (0.63-0.85) | <0.001 | | 46.5 | | 0.044 | |  |
|  | **Percentage III/IV gastric cancer** | | | | | | | | |
|  | ≥ 90% | 0.75 (0.55-1.02) | 0.069 | | 49.1 | | 0.097 | | 0.497 |
|  | < 90% | 0.67 (0.56-0.79) | <0.001 | | 47.8 | | 0.053 | |  |
|  | **Follow-up duration (years)** | | | | | | | | |
|  | ≥ 5 years | 0.75 (0.66-0.85) | <0.001 | | 39.0 | | 0.119 | | 0.069 |
|  | < 5 years | 0.55 (0.38-0.80) | 0.002 | | 43.7 | | 0.114 | |  |
